# Harnessing a decade of research at the Research Institute for Bioscience and Biotechnology in Kathmandu, Nepal: Proceedings of the Fourth International Conference ICBB-2022

**DOI:** 10.1186/s12919-023-00252-3

**Published:** 2023-06-02

## Suvechhya Bastola^1^, Remco Kort^2^, Prajwal Rajbhandari^1^

### ^1^Department of Applied Microbiology and Food Technology, Research Institute for Bioscience and Biotechnology, Kathmandu, Nepal; ^2^Amsterdam Institute for Life and Environment (A-LIFE), Vrije Universiteit Amsterdam, Amsterdam, The Netherlands

#### **Correspondence:** Prajwal Rajbhandari (prajjwalrajbhandari@ribb.org.np)

*BMC Proceedings 2023*, **17(Suppl 3):**

**Introduction**

The Federal Democratic Republic of Nepal is a relatively small country of approximately 150,000 km^2^, but extraordinarily rich in natural resources, including lush plains, hilly woodlands, and eight of the world's ten tallest mountains. The altitudes in Nepal vary from less than 60 m in the lowlands of Terai in the South to the crest of the Himalaya reaching 8848 m in the North. The geographic variations and the latitude gradient, all contribute to the species richness in this region of the world. Nepal represents an important knowledge center regarding the use of various plant species in the form of traditional medicines [1]. However, the majority of the bioresources are poorly characterized and inadequately documented. The scientific applications are numerous: extracts from medicinal plants, and bioactive compounds from soil microbes could serve as antibiotics, anticancer drugs, and nutraceuticals in modern day medicine. Apart from their use in applied medicinal research, natural resources provide in a broad sense invaluable ‘ecosystem services’. These services encompass varied benefits to humans provided by the natural environment, including the provision of food, clean drinking water, and the decomposition of waste. Documented research has become essential in preserving these natural resources. The urgent need for exploration of nations' valuable biodiversity is a major driver for the foundation of the Research Institute for Bioscience and Biotechnology (RIBB).

The RIBB was founded in 2011 with the mission to advance the fields of bioscience, engineering, innovation and education by collaborating with academic and other research organizations to conduct high quality research in a world-class academic and professional environment. The RIBB is now one of the leading non-profit research organizations in the field of biotechnology as it conducts high quality research forming an integrated part with the academia in Nepal. The country’s rich natural resources form an essential part of the RIBB’s research focus, as the institute started to explore and study indigenous medicinal plants and soil microbes from the first years after its foundation. Notably, we were the first research group that characterized the genome of the actinobacteria *Nonomuraea* [2] and *Pseudonocardia* [3] isolated from a Nepalese mud dauber wasp Nest. Furthermore, we described the molecular structures of non-toxic antioxidant compounds from endophytic fungus *Preussia* [4] and the soil bacterium *Streptomyces* [5]. In pursuit of exploring new compounds originating from Nepalese rich biodiversity, we extracted natural antimicrobial and antioxidant compounds for applications as edible coating on fruits and vegetables [6], and encapsulation of essential oils inside hydrogels [7].

RIBB is also addressing local community problems like water pollution and sanitation hazards, which have developed as a consequence of urbanization. Our research carried out in collaboration with Newcastle University, shows how inadequate wastewater management led to public health risks [8]. Similarly, Phutung Research Institute (PRI, Kathmandu, Nepal) in collaboration with University of York and RIBB is developing a portable low-cost Water Assessment System (WAS), which will allow the early detection of fecal contamination [9]. However, to be able to valorize its research efforts, collaborative and multidisciplinary research is essential. Therefore, RIBB has been organizing the “International Conference on Bioscience and Biotechnology” (ICBB) biannually from 2016. ICBB aims to provide a platform for students, established researchers, entrepreneurs, other relevant stakeholders from Nepal and around the world to convene and discuss progress and collaboration on various topics related to bioscience and biotechnology. In the disparate settings of Nepal, ICBB not only focused on the capital city, but rotated among other cities outside Kathmandu in order to incorporate a new audience and research opportunities. The first edition of the conference, the ICBB-2016, was held in Kathmandu with natural products as its major theme. The second edition, the ICBB-2018, took place in Dhulikhel with the focus on biobanking and translational medicine. This was followed up by the third edition, the ICBB-2020, in Pokhara about food and applied microbiology. The fourth edition, the ICBB-2022, entitled ‘A decade in research: Celebrating RIBB’s 10th anniversary’, encompassed a wider variety of research themes. This meeting marked a year-long of various formal and informal events, celebrating the institute's ten years existence on 4^th^ of July 2021.

The ICBB-2022 meeting was jointly organized by the RIBB and University of Nepal Development Board (UoN-DB). It was co-organized by PRI, Kathmandu Research Institute for Biological Sciences (KRIBS) and Engage Nepal with Science (ENwS). The conference spanned four days and featured Plenary lectures, Invited talks, Oral and Poster presentations. The five main sessions included Session I: Traditional Fermented Food and Nutritional Science; Session II: Biomedicine and Translational Research; Session III: Protein Science and Its Application; Session IV: Water, Wildlife and Environment; Session V: Graduate Student Talks. The meeting was conducted in a hybrid model, where scientists and researchers from 11 countries with 126 in-person participants and more than 500 zoom links were distributed to share scientific knowledge from their field of expertise as well as to celebrate RIBB’s journey of success, challenges and accomplishments.

The first day of the conference was dedicated to two workshops organized in parallel, followed by introductory speeches from the organizers and co-organizers. The plenary session on the second day was conducted by Dr. Max Paoli and Payel Patel from The World Academy of Sciences (TWAS), Trieste, Italy. Similarly, the Plenary session on the third day was conducted by Dr. Alba Abad from University of Edinburgh, UK (Table 1).

The proceedings ICBB-2022 included a total of 32 abstracts. These abstracts covered a wide range of research topics under each thematic session. The first session covered probiotics, an emerging concept in the field of functional foods, fermentations for the degradation of mycotoxins, and Nepalese traditional natural fermentations, including nutritional perspectives. The second session covered leading innovative technologies and approaches in the field of biomedicine like antiviral immunity in the case of SARS-COV-2 and precision medicine to control neglected tropical diseases. Metagenomics and Next Generation Sequencing (NGS) in the context of public health in Nepal was also presented in this session. The third session included talks in the water, wildlife and environment including recent topics like tiger genetics carried out in Nepal. The fourth session on protein science covered some recent developments in this field, related to protein-protein interactions, the role of SUPT5H in breast cancer, and engineered living material. Finally, the floor was opened to recent graduates, representing a new generation of Nepalese researchers, for their short talks on their theses and poster presentations, including promising research topics like biopesticides, natural priming of seeds, low-cost polymerase chain reaction technology, and valorization of waste materials.

Since 2016, the Research Institute for Bioscience and Biotechnology (RIBB) has been able to successfully organize the International Conference on Bioscience and Biotechnology (ICBB) every two years, which has grown to be an event of international resonance. This conference plays a critical step in creating an international platform for attendees to connect with international partners, collaborate on local projects and thus get opportunities to access various expertise and facilities, through networking. As RIBB reached its milestone of completing 10 years in the field of research, this 4^th^ edition of ICBB was dedicated to appreciating its prosperous journey. This platform provided an opportunity to reflect on the institute’s past invaluable learnings and successes, and further explore future possibilities of growth and excellence in the field of bioscience and biotechnology. In these years, RIBB has been able to form a consortium of committed, highly trained and competitive group of 15 young principal investigators, more than 100 undergraduate and graduate researchers, and leaders in today’s interdisciplinary and dynamic research environment. The Institute also took this opportunity to acknowledge the contributions of all those who had supported along the way and made the journey possible. Thereupon, 18 inductees for the institute’s RIBB Hall of Fame were honored at the closing dinner gathering.

Furthermore, a new session (Water, Wildlife and Environment) was introduced to acknowledge the importance of the conference environment (Sauraha, a renowned wildlife sanctuary of Nepal). This session included cutting edge technologies used in environmental research and biodiversity conservation in Nepal, opening ways to push interdisciplinary research activities among local research institutes to mitigate environmental and biodiversity challenges. Similarly, ICBB-2022 also introduced the Graduate Student Talks Session, which provided a platform for university students to present their research outcomes to a broader audience including international scientists. The workshop on Public Engagement with school students gave the local students exposure to hands-on scientific activities and provided them an opportunity to interact with local scientists and researchers. Along with that, the workshop also supported their confidence in Science, Technology, Engineering and Maths (STEM). The second workshop on WAS demonstrated water quality research to the general public and stakeholders of local communities, also spreading awareness on water pollution. In addition, discussions at the conference led to agreement for an overarching research topic and location for ICBB-2024. The next meeting will take place in Godawari, Lalitpur on the urgent theme of "Planetary Health”, planned as a satellite meeting of the 2024 Planetary Health Annual Meeting (PHAM) hosted by the Sunway Centre for Planetary Health in Malaysia. The ICBB-2022 successfully integrated and provided exposure for the local scientific members residing outside of the capital to international as well as national researchers. As organizers, we are immensely pleased that we were able to continue this conference to its fourth edition. We take special merit of ICBB-2022 as it was organized amidst the uncertainties of the COVID-19 pandemic in a situation where the world and specially the scientific sector in Nepal was badly shaken and struggling to recover.

**References**

1. Kunwar RM, Bussmann RW. Ethnobotany in the Nepal Himalaya. Journal of Ethnobiology and Ethnomedicine. 2008 Dec;4(1):1-8.
2. Aryal N, Aziz S, Rajbhandari P, Gross H. Draft genome sequence of *Nonomuraea* sp. strain C10, a producer of brartemicin, isolated from a mud dauber wasp nest in Nepal. Microbiology Resource Announcements. 2019 Nov 7;8(45):e01109-19.
3. Aryal N, Aziz S, Rajbhandari P, Gross H. Draft Genome Sequence of the Sattazolin-Producing Strain *Pseudonocardia* sp. C8, Isolated from a mud dauber wasp nest in Nepal. Microbiology Resource Announcements. 2021 Mar 11;10(10):e00007-21.
4. Paudel B, Bhattarai K, Bhattarai HD. Antimicrobial and antioxidant activities of two polyketides from lichen-endophytic fungus *Preussia* sp. Zeitschrift für Naturforschung C. 2018 Mar 1;73(3-4):161-3.
5. Paudel B, Maharjan R, Rajbhandari P, Aryal N, Aziz S, Bhattarai K, Baral B, Malla R, Bhattarai HD. Maculosin, a non-toxic antioxidant compound isolated from *Streptomyces* sp. KTM18. Pharmaceutical Biology. 2021 Jan 1;59(1):931-4.
6. Majhi R, Maharjan R, Shrestha M, Mali A, Basnet A, Baral M, Duwal R, Manandhar R, Rajbhandari P. Effect of altitude and solvent on *Psidium guajava* Linn. leaves extracts: phytochemical analysis, antioxidant, cytotoxicity and antimicrobial activity against food spoilage microbes. BMC chemistry. 2023 Dec;17(1):1-6.
7. Shakya N, Budha Chettri S, Joshi S, Rajbhandary A. Utilization of FMOC-3F-PHE hydrogel for encapsulation of *Zanthoxylum armatum* and *Cinnamomum camphora* oil for enhancing their antibacterial activity. BMC research notes. 2022 Dec;15(1):1-6.
8. Pantha K, Acharya K, Mohapatra S, Khanal S, Amatya N, Ospina-Betancourth C, Butte G, Shrestha SD, Rajbhandari P, Werner D. Faecal pollution source tracking in the holy Bagmati River by portable 16S rRNA gene sequencing. NPJ Clean Water. 2021 Feb 18;4(1):1-0.
9. Bohora S, Li K, Waiba P, Gautam S, Martins A, Rodrigues RA, Kikstra P, van der Horst M, Martins ER, Krauss TF, Dhakal A. A Low-cost Fresnel Lens Fluorometer to Detect Fecal Contamination in Drinking Water in Realtime. InCLEO: Applications and Technology 2022 May 15 (pp. AM5M-8). Optica Publishing Group.

**Table 1.**
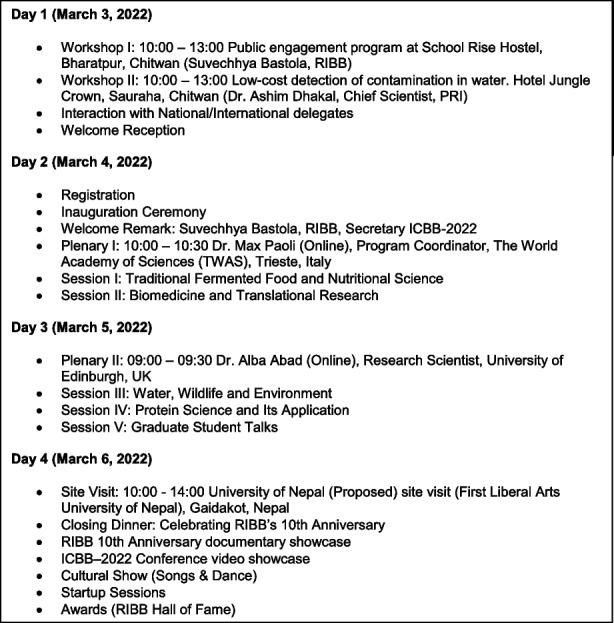
Outline of the conference program of ICBB-2022, Sauraha, Chitwan, Nepal.

## A1. Workshop on public engagement program at school

### Suvechhya Bastola^1,2^, Alba Abad^1,3^, Rojlina Manandhar^4^, Surakshya Singh^4^, Bibek Chandra Mahaseth^4^, Kamana Dawadi^4^, Lochan Pandeya^4^

#### ^1^ Engage Nepal with Science, Edinburgh, UK; ^2^ Learning Lab, Research Institute for Bioscience and Biotechnology, Kathmandu, Nepal; ^3^ Wellcome Centre for Cell Biology, University of Edinburgh, Edinburgh, UK; ^4^ Department of Natural Products and Green Chemistry, Research Institute for Bioscience and Biotechnology, Kathmandu, Nepal.

##### **Correspondence:** Suvechhya Bastola (suvechhya.bastola@ribb.org.np)

*BMC Proceedings 2023*, **17(Suppl 3):**A1

**Background**

Engage Nepal with Science runs as collaboration between the University of Edinburgh (United Kingdom) and the Research Institute for Bioscience and Biotechnology (Nepal) and aims to spread the culture of engaging with science to empower, inspire and build confidence in STEM. We use gamification and ‘learning-by-doing’ as tools to promote engagement and sustained motivation in learning for both students and teachers. Our network of school teachers and RIBB researchers work together towards making science learning more creative and dynamic following the school curriculum.

**Methods**

The hands-on science workshop (that included a group demonstration for soap making along with activities on plant/animal cells, microbes and algae) was organized at School Rise Hostel, Chitwan. 40 students from 7-15 years old and 3 teachers attended the workshop.

**Results**

Through the soap making demonstration, the students were made aware of the microbial presence in our hands and the importance of hand-washing. Preparation of slides of plant/animal cells was an opportunity to have first-hand experience on microscope handling. Our *A zoo in your pond* activity allowed the students to observe different samples of water under the microscope. Our *Make your own microbe!* made students aware of the different features of microbes and the role of those microbes in different human diseases. The microalgae activity replicated the laboratory setup to understand that algae can be used to our advantage to produce iron pills. Practical-based learning enticed students to become curious and were encouraged to think in a scientific, evidence-based way.

**Conclusion**

Hands on based learning for the students, makes the learning process more enjoyable and long-lasting. The activities inspire students, boost their creativity and support their science learning. These workshops also inspire teachers, build their confidence in science communication and support their science communication skills to make learning of science subjects at schools more creative and dynamic.

## A2. Workshop on low-cost and real-time water assessment system (WAS) for the detection of fecal contaminants in water

### Anusa Thapa^1^, Rijan Maharjan^1^, Pravin Bhattarai^1^, Suvechhya Bastola^1,2^, Sishir Gautam^1^, Sanket Bohora^1^, Prashant Waiba^1^, Kezheng Li^3^, Augusto Martins^4^, Ricardo A. Rodrigues^5^, Peter Kikstra^5^, Marcel van der Horst^5^, Prajwal Rajbhandari^2^, Emiliano R. Martins^4^, Thomas Krauss^3^, Ashim Dhakal^1^

#### ^1^Biophotonics Lab, Phutung Research Institute, Tarakeshor-7, Kathmandu, 44611, Nepal; ^2^Department of Applied Microbiology, Research Institute for Bioscience and Biotechnology, Kathmandu, Nepal; ^3^Department of Physics,University of York, York YO10 5DD, UK; ^4^São Carlos School of Engineering, Department of Electrical and Computer Engineering, University of São Paulo, 13566-590, Brazil; ^5^Amsterdam Sensor Lab, Amsterdam University of Applied Sciences, 1097 Amsterdam, The Netherlands

##### **Correspondence:** Ashim Dhakal (ashim.dhakal@pinstitute.org)

*BMC Proceedings 2023*, **17(Suppl 3):**A2

**Background**

To address the lack of real-time and low-cost methods to detect fecal contamination in drinking and household water needed in resource-limited settings, we have developed a Water Assessment System (WAS). WAS is based on a fluorometry of biochemicals emitted by active organisms in fecal matter. WAS has a user-friendly interface which provides the users the level of fecal contamination in water via Traffic light indicators, and also the measured level of contamination. We organized this workshop to survey any existing tools potential users are using, their disposition towards using our current WAS model, the price they are willing to pay, and any other features they would need.

**Materials and methods**

A total of 56 participants from regulatory agencies such as community managed water suppliers, live-stock farmers, food and beverage sector, environmental sector and general consumers attended this workshop where we had a hands-on demonstration of our prototype for WAS.

**Results**

94% of participants for household use and 83% from the commercial sector stated that they would want to own a WAS device. 50% of participants were not aware of technologies to test their water in spite of experiencing water-borne diseases every year. While the regulatory agencies participants indicated WAS will be useful, only 56% of these participants wanted to pay for the current version because they already had their own labs for testing water. 57%, 27% and 10% of the participants respectively stated they are willing to pay <50 USD, <100 USD, <250 USD for the current model. However, industrial participants were willing to pay as much as $3,000 if automatic and continuous monitoring was implemented in the current model. More technical users wanted higher specificity to detect certain pathological organisms and harmful ions. While the less technical users indicated that the device display interface should have nepali scripts.

**Conclusion**

WAS has a good market in the commercial and environmental sector, such as, live-stock farming, food and beverage industry, drinking water suppliers, and wildlife conservation. If we are able to sell at a cost < 250 USD, there is a good market for household users as well.

**Acknowledgement**

We thank the Diyalo Foundation for their support in developing this technology. This work was supported by EPSRC’s GCRF Grant# EP/T020008/1 & EP/W524165/1; TWAS & SIDA Grant # 18-013.

## A3. RIBB’s 10^th^ year journey: story presentation

### Prajwal Rajbhandari

#### Department of Applied Microbiology and Food Technology, Research Institute for Bioscience and Biotechnology, Kathmandu, Nepal

##### **Correspondence:** Prajwal Rajbhandari (prajjwalrajbhandari@ribb.org.np)

*BMC Proceedings 2023*, **17(Suppl 3):**A3

**Background**

Inception of the Research Institute for Bioscience and Biotechnology (RIBB) started at the Department of Biotechnology, Kathmandu University in 2008 (Fall). However, foundation as a research institute was placed on 4^th^ of July, 2011 at Sinamangal, Kathmandu. We started an institute office with a floor size of 600 sq. feet for writing grants and received our first grant on April 30, 2013, which was for organizing a workshop on dairy waste management. From there onwards we never looked back. Before celebrating the institute's 5^th^ anniversary, we had already received a few research grants that supported establishing a laboratory for natural product chemistry and soil microbiology to explore Nepalese biodiversity.

After completing 5 years of journey, we decided to provide basic laboratory facilities (equipment and consumables) and working environment to Nepalese scientists and researchers who returned back to Nepal and wanted to continue their research journey. This initiative not only helped the institute grow in broader areas related to bioscience and biotechnology, but also helped train young minds in research before heading for graduate studies. Along with that, we also established two new laboratories to start basic science research in the field molecular biology and cell culture at our second location, in Nakkhu. Looking back on its glorious 11 years, RIBB has been able to establish a sound research culture, collaborated with more than 10 European laboratories, received competitive European funding where 15 young principal investigators started their research career, opened 40 plus job opportunities and trained more than 100 undergraduate and graduate students through research internships and thesis.

Above all, three members of the Nepal Research Alliance (NRA), namely Kathmandu Research Institute for Biological Sciences, Phutung Research Institute and RIBB envisioned a Research Campus named “Biomedicum” which is now initiated at Balkumari to kickstart interdisciplinary research, where scientific expertise, equipment and other common resources are shared under one roof to push research ecosystem in Nepal.

**Acknowledgement**

As a research institute, we would like to thank our funding agencies (Korea Green Foundation, RenewableNepal Programme, TWAS, IFS, Alexander von Humboldt Foundation, Elsevier Foundation, SfAM, NHRC, NAST, CIM-GIZ, OWSD and British Council) for their support to build a totally new research institute in the field of biotechnology from scratch in Nepal.

## A4. Biomedicum: Need for “ecosystem thinking” for development of R&D in Nepal

### Ashim Dhakal

#### Biophotonics Lab, Phutung Research Institute, Tarakeshor-7, Kathmandu, 44611, Nepal

##### **Correspondence:** Ashim Dhakal (ashim.dhakal@pinstitute.org)

*BMC Proceedings 2023*, **17(Suppl 3):**A4

Nepal lacks basic elements of a functional innovation, research and development (R&D) ecosystem, such as, funding agencies for R&D activities, legal framework to recognize research institutes or incentivize R&D activities, organized R&D peer-groups, R&D infrastructure, R&D supply chain and R&D human resources. Existing R&D bodies seem to be inefficient and inadequate. Recognizing this need, a handful of young researchers trained elsewhere returned to Nepal with initiative to establish non-governmental and nonprofit research institutes. Eight of those research institutes viz. Annapurna Research Center, Center for Health and Disease Studies- Nepal, Global Institute for Interdisciplinary Studies, Kathmandu Institute of Applied Sciences, Kathmandu Research Institute for Biological Sciences (KRIBS), Nepal Applied Mathematics and Informatics Institute for Research, Phutung Research Institute (PRI) and Research Institute for Bioscience and Biotechnology (RIBB) allied themselves informally as *Nepal Research Alliance* with broad and collective objectives to facilitate collaboration between the themselves, and to formulate a common view-point for recommending Nepal government appropriate policies in order to advance scientific and technological research in Nepal. Thanks to lobbying efforts of the NRA, Nepal Government took initiative to draft and promulgate National Science, Technology and Innovation Policy- 2019 with direct involvement of NRA members. However, the pandemic, and government changes, changes in the leadership in the Ministry of Science and Technology slowed meaningful implementation of the policy. There is a need to revive the activism among the researchers for the development of the R&D ecosystem in Nepal.

However, in spite of the several hurdles during the COVID-19 pandemic, three members of the NRA, namely KRIBS, PRI and RIBB signed a collaboration agreement to come together in a single Research Campus called “Biomedicum”, and share scientific expertise, equipment and other resources. This collaboration has resulted in three collaborative research and co-publications. It exemplifies a collaborative approach and “ecosystem thinking” for development of R&D in Nepal.

## A5. University of Nepal – programs & master plan presentation

### Arjun Karki

#### University of Nepal (UoN) Development Board, Kathmandu, Nepal

##### **Correspondence:** Arjun Karki (arjun.karki@uon.edu.np)

*BMC Proceedings 2023*, **17(Suppl 3):**A5

There is a widespread perception and belief that the existing model and practice of higher education in Nepal has not adequately responded to the existing needs and the emerging challenges of the nation. That has also contributed to the exodus of young Nepali students to foreign countries, most of whom get lost there. The proposed University of Nepal (UoN) is an initiative undertaken to address that very issue. The UoN is envisaged to be a top quality, interdisciplinary, research oriented, and autonomous public University in Nepal. During the last five years since the project was initiated, remarkable progress has been made. That includes, but not limited to, defining the overarching conceptual framework for the UoN, academic program planning, engagement and collaboration with the local governments in Nawalparasi, located about 200 Kilometers South-West of Kathmandu, to avail the land for building the UoN Campus. In addition, the government of Nepal has formed the UoN Development Board (DB) with the mandate to undertake all the preparatory work necessary for the establishment of the UoN and launching of its academic programs.

The UoN aims to prepare graduates who are intellectually competent, conscientious, creative, willing to embrace the spirit of citizenship and demonstrate the courage to play a leadership role to bring about positive and transformative changes in Nepal. In order to achieve that goal, the UoN envisages running the liberal arts and sciences based undergraduate programs that helps students not only to better understand themselves and the world around them but also to acquire the critical thinking, communication and problem-solving skills. Graduate programs will also be introduced once the needed infrastructure and faculty strength is consolidated. In addition to knowledge dissemination, new knowledge generation (i.e. research) will be an integral part of the UoN life. In pursuing its mission, the UoN intends to learn and benefit from the relevant global and regional experiences and best practices. It therefore seeks to engage with like- minded partners for the purpose of institutional capacity development as well as the promotion of academic and research collaboration in the time ahead.

## A6. Fermentation as a novel approach for the prevention of aflatoxins induced liver cancer

### Paul Alex Wacoo^1^, Simon Aliker^2^, Andrew Bukenya^1^, Deborah Wendiro^3^, Joseph F Hawumba^2^ and Ponsiano Ocama^4^

#### ^1^Department of Medical Biochemistry, School of Biomedical Sciences, College of Health Sciences, Makerere University, P.O. Box 7062 Kampala, Uganda; ^2^Department of Biochemistry and Sports Science, School of Biological Sciences, College of Natural Sciences, Makerere University, P.O. Box 7062 Kampala; ^3^WENA Biosciences Institute P.O. Box 26, Kamuli, Ugand; ^4^Department of Internal Medicine, School of Medicine, College of Health Sciences, Makerere University, P. O. Box 7062 Kampala

##### **Correspondence:** Paul Alex Wacoo (wacooalex@gamil.com)

*BMC Proceedings 2023*, **17(Suppl 3):**A6

**Background**

Aflatoxins-induced liver cancer is a hidden public health hazard requiring immediate action. Particularly in sub-Saharan Africa and East Asia where 83% of the new cancer cases have been reported due to synergistic contribution between aflatoxins and highly endemic hepatitis B infection [1]. Aflatoxins are associated with the food chain right from production to post consumption. A novel strategy to decontaminate aflatoxins before and after feeding is the best hope for mitigating the risk effect of aflatoxin. Probiotic bacteria have been demonstrated to mitigate the risk effect of aflatoxin in food [2] and after ingestion thus providing protection against aflatoxicosis and liver cancer and also ensure access to nutritious, safe and healthy food.

**Materials and methods**

A case-controlled design was used to evaluate the association between aflatoxin and liver cancer. The questionnaire collected demographic information and frequency of consuming aflatoxin high risk foods. Urine collected and urinary aflatoxin M1 analyzed as described by Wacoo et al., [3]. Stools collected and microbiota analysis was done according to Atukunda et al., [4]. Lactic acid bacteria were isolated from stool samples as described by Wacoo et al., [3]

**Results**

The odds of developing liver cancer were four times higher in the case group than in the control group. Mean urinary aflatoxin M1 levels were generally higher in the case group than in controls. The risk of developing liver cancer (odds ratio) decreased with decrease in the frequency of consumption of maize bread/porridge. Generally, the abundance of *Lactobacillus* spp. in the case group was higher than in controls. However, levels of *Lactobacillus* spp. in control were inversely proportional to urinary aflatoxin M1 levels. *Lactobacillus* spp. in Case groups showed no observable relationship with urinary aflatoxin M1 levels. Approximately, 90% out of 400 of the *Lactobacillus* isolates demonstrated potential to adsorb aflatoxin. Since, the earlier study demonstrated the potential of *Lactobacillus rhmanosus* in decontamination of aflatoxin B1 in maize porridge [5], more experiments are being done to screen the isolates for aflatoxin degradation in maize porridge and other food types.

**Conclusions**

The risk of liver cancer is directly proportional to the frequency of consumption of contaminated maize bread/porridge. The range of *Lactobacillus* spp in controls is inversely correlated to urinary aflatoxin M1 levels. Lactobacillus spp showed great potential to remove aflatoxin from maize porridge.

**Acknowledgements**

This work was funded by the Government of Uganda through the Makerere University Research and Innovations Fund (Mak-RIF). The authors acknowledge the assistance of Andrew Bukenya, Simon Aliker, Imelda Namirembe and Caroline Mutesi during participant recruitment, sample collection and analysis.

**Consent to publish**

The study protocol conforms to the ethical guidelines of the 1975 Declaration of Helsinki (6th revision, 2008) as reflected in a priori approval by the Research Ethics committee of School of Medicine, College of Health Sciences, Makerere University (# REC REF 2020-083) and by Uganda National Council for Science and Technology (no. UNCST HS707ES).

**References**

1. Wu F: The global burden of disease caused by foodborne aflatoxin. *WHO Commissioned Report Geneva: World Health Organization Food Borne Disease Burden Epidemiology Reference Group* 2010.
2. Ahlberg SH, Joutsjoki V, Korhonen HJ: Potential of lactic acid bacteria in aflatoxin risk mitigation. *International journal of food microbiology* 2015, 207:87-102.
3. Wacoo AP, Atukunda P, Muhoozi G, Braster M, Wagner M, Van Den Broek TJ, Sybesma W, Westerberg AC, Iversen PO, Kort R: Aflatoxins: occurrence, exposure, and binding to Lactobacillus species from the gut microbiota of rural Ugandan children. *Microorganisms* 2020, 8(3):347.
4. Atukunda P, Muhoozi GK, van den Broek TJ, Kort R, Diep LM, Kaaya AN, Iversen PO, Westerberg AC: Child development, growth and microbiota: follow-up of a randomized education trial in Uganda. *Journal of global health* 2019, 9(1).
5. Wacoo AP, Mukisa IM, Meeme R, Byakika S, Wendiro D, Sybesma W, Kort R: Probiotic enrichment and reduction of aflatoxins in a traditional African maize-based fermented food. *Nutrients* 2019, 11(2):265.

## A7. Nutritional perspective and its consequences in Nepal

### Keshab Bhattarai, Sudhashree Adhikari

#### Sattwik Nutri-Food (a Diet Therapy and Research Center), Pokhara, Nepal

##### **Correspondence:** Keshab Bhattarai (drbhattaraikeshab@gmail.com)

*BMC Proceedings 2023*, **17(Suppl 3):**A7

**Background**

Nutritional and healthy foods are the basic requirements for the people around the globe to stay healthy and prevent diseases. Nepal as a geographically diversified country with many staple foods are grown. With the development of technology and a busy schedule of life, risk of having chronic diseases are increasing day by day. Globally, adults after the age of 30s are suffering from different types of chronic diseases and the root cause of these diseases are unhealthy food habits.

**Case report**

Different data shows that chronic diseases are a leading cause of death globally with 70% of global deaths after 2015. Diabetes is the fourth after cardiovascular disease, cancer and respiratory diseases and is responsible for 1.6 million deaths globally. Out of total deaths worldwide, 80% of global diabetes deaths occur in low-income countries due to lack of awareness, undiagnosed and unhealthy food habits. Low income South Asian countries, such as Nepal, have seen a particularly rapid increase in the prevalence of diabetes in the past two decades. Now it is the time to educate people about the importance of optimal nutrition, food, and lifestyle in managing dietary disorders. Over-consumption and under-consumption of macro and micronutrients can cause various diseases. For example, protein deficiency can result in anemia, but the over intake can place more stress on the kidneys that could lead to a cause of renal deterioration. Similar cases were identified in the over or under consumption of different nutrients. From the identified issues above, solving the problem manually based on human decision is difficult and tedious.

**Conclusion**

Many studies have shown that changing eating behavior and lifestyle can lower the risk of having chronic diseases. The mission of the Sattwik Nutri-Food a Diet Therapy and Research Center is to motivate the people towards changing their lifestyle by providing customized food solutions with locally grown products with modern flavor of cooking and preparation. It has a mission to create an “environment of sustainable food system” that can create a chronic disease-free society.

## A8. Microbial composition of milk fermented by designed and natural starter cultures

### Remco Kort^1,2,3^ and Wilbert Sybesma^3^

#### ^1^A-Life, Vrije Universiteit Amsterdam, The Netherlands; ^2^ARTIS-Micropia, Amsterdam, The Netherlands; ^3^Yoba for Life foundation, Amsterdam, The Netherlands

##### **Correspondence:** Remco Kort (r.kort@vu.nl)

*BMC Proceedings 2023*, **17(Suppl 3):**A8

**Background**

Clinical studies have shown that oral administration of the probiotic strain *Lactobacillus rhamnosus* GG, a human intestinal isolate, reduces the duration of acute infectious diarrhea, including rotavirus-associated diarrhea [1]. This study aims to elucidate the microbial composition of a variety of fermented milks and investigate the fermentative capacity of a designed starter culture serving as a vehicle allowing the propagation of this probiotic bacterium to confer intestinal health benefits.

**Materials and methods**

We have designed a novel dried starter culture that surmounts the inability of the probiotic model strain *Lactobacillus rhamnosus* GG to grow in milk.

**Results**

We have formulated a dried starter culture based on a proteolytic strain of *Streptococcus thermophilus* that enables the propagation of both strains in milk and other food matrices, including soy, millet and maize [2]. The affordable starter culture is currently used by people in resource-poor communities in Africa to ferment cow’s milk and cereals [3]. We have demonstrated that by using this starter culture, we could integrate health-promoting probiotics into a number of fermented food products. Hereto, we characterized the bacterial community composition of a naturally fermented milk product (lait caillé) from northern Senegal, prepared in wooden bowls (lahals) containing bacterial biofilm that serves as an inoculum for the fermentation process [4]. In addition, we have incorporated our probiotic starter culture containing *Lactobacillus rhamnosus* GG into the local fermentation process and demonstrated growth of this probiotic bacterium in several different food matrices [5].

**Conclusion**

We have set up an efficient and practical delivery method for probiotic bacteria in fermented foods by designing a novel starter culture with the potential to confer additional health benefits.

**References**

1. Szajewska H, Kołodziej M, Gieruszczak-Białek D, Skórka A, Ruszczyński M, Shamir R. Systematic review with meta-analysis: *Lactobacillus rhamnosus* GG for treating acute gastroenteritis in children - a 2019 update. Aliment Pharmacol Ther. 2019; 49:1376-1384.
2. Kort R, Westerik N, Mariela Serrano L, Douillard FP, Gottstein W, Mukisa IM, Tuijn CJ, Basten L, Hafkamp B, Meijer WC, Teusink B, de Vos WM, Reid G, Sybesma W. A novel consortium of *Lactobacillus rhamnosus* and *Streptococcus thermophilus* for increased access to functional fermented foods. Microb Cell Fact. 2015; 14:195
3. Reid G, Sybesma W, Matovu W, Onyango A, Westerik N, Kort R. Empowering women through probiotic fermented food in East Africa. J Glob Health. 2020; 10:010330.
4. Parker M, Zobrist S, Donahue C, Edick C, Mansen K, Hassan Zade Nadjari M, Heerikhuisen M, Sybesma W, Molenaar D, Diallo AM, Milani P, Kort R. Naturally fermented milk from Northern Senegal: Bacterial community composition and probiotic enrichment with *Lactobacillus rhamnosus*. Front Microbiol. 2018; 9:2218.
5. Wacoo AP, Mukisa IM, Meeme R, Byakika S, Wendiro D, Sybesma W, Kort R. Probiotic Enrichment and Reduction of Aflatoxins in a Traditional African Maize-Based Fermented Food. Nutrients. 2019; 11:265.

## A9. Introducing cutting-edge technology to prevent and control neglected tropical diseases: how far we are?

### Braulio M. Valencia

#### Viral and Immunology Systems Program (VISP), Kirby Institute, UNSW, Australia

##### **Correspondence:** Braulio M. Valencia (barroyo@kirby.unsw.edu.au)

*BMC Proceedings 2023*, **17(Suppl 3):**A9

**Background**

Precision medicine, targeted therapy, or next-generation sequencing have been restricted to chronic communicable or non-communicable conditions mostly prevalent in developed countries. Technology improvement has considerably reduced costs and complexity, making cutting-edge technologies potentially accessible for low/middle-income settings and applicable to diverse medical conditions, including neglected tropical diseases. In this presentation, examples of cutting-edge applications and their applicability as standards of care in preventing and controlling selected neglected tropical diseases will be discussed.

**Materials and methods**

A PubMed search including “point-of-care” and “infectious diseases” was conducted to identify cutting-edge and affordable technologies to diagnose infectious diseases and characterise biomarkers of severity and outcome.

**Results**

Studies were mostly based on nucleic acid detection. Despite sample processing and duration were reported as considerably improved, these were pathogen-specific or restricted to locally prevalent pathogens. Few publications tested targeted diagnostic approaches for prevalent pathogens causing syndromic conditions (i.e. acute respiratory infections, STDs, acute gastro-enterocolitis). Only metagenomics was tested as an open-ended diagnostic approach assessed in syndromic conditions as community-acquired pneumonia and meningitis, endophthalmitis, diabetic foot samples, and sepsis. Accuracy and effectiveness, however, showed broad variability depending on the clinical specimen (i.e. respiratory secretions vs. whole blood) and the prevalent pathogens (i.e. bacterial vs. viral). Compared to other nucleic acid tests, metagenomics also demonstrated usefulness in identifying biomarkers of severity or treatment response as antimicrobial resistance or transcriptomic markers of severity. Depending of the specimen type, metagenomics is also suitable to identify host gene variants (i.e. IFN lambda 4 and direct-acting antivirals) previously associated with adverse outcomes.

**Conclusions**

Including metagenomics as a standard of care is feasible given its diverse applicability on diagnostics, biomarkers, and prognostic purposes. More investigation is required to improve nucleic acid recovery and threshold detection specially for viral pathogens and in heavily contaminated samples or with abundant host background.

## A10. Metagenomic next generation sequencing and its utilization in context of public health in Nepal

### Nishan Katuwal^1^, Rajeev Shrestha^1,2^

#### ^1^Molecular and Genome Sequencing Research Lab, Dhulikhel Hospital, Kathmandu University Hospital, Dhulikhel, Kavre, Nepal; ^2^Department of Pharmacology, Kathmandu University School of Medical Sciences, Dhulikhel, Kavre, Nepal

##### **Correspondence:** Nishan Katuwal (nishankatuwal@dhulikhelhospital.org)

*BMC Proceedings 2023*, **17(Suppl 3):**A10

**Background**

Metagenomic Next Generation Sequencing (mNGS) is a sequencing approach that allows to comprehensively sample all genetic material present in a given complex sample and compare the genetic material to a global database of all known pathogens, to identify which organisms are present. There are various applications to mNGS in diagnosing infectious diseases, outbreak tracking, infection surveillance, pathogen discovery and many more.

**Discussion**

In the context of health, the identification of the causative agent behind any illness is important for developing effective clinical management strategies. However, the characterization of complex infections possess tremendous burden as such infections are difficult to culture and investigate in underdeveloped infrastructures. Additionally, the employment of molecular methods, such as PCR, require prior genetic information on the causative agents, for developing specific primers for detection. Metagenomic Next Generation Sequencing approach, on the other hand, can parallelly identify minute amounts of infections and coinfections, of varying origin in a single investigation. Instead of performing multiple targeted assays, each looking for a specific pathogen, metagenomic approach uses a single sequencing-based method that is capable of identifying most, if not all, microorganisms. It is a very sensitive and rapid method that can promptly assist the selection of treatment regimens for the disease.

Additionally, metagenomics has helped to elucidate strong correlation between antimicrobial resistance and microbiome through discovery of complex microbial communities and their components involved in antimicrobial resistance. It can also be utilized for identification of novel antimicrobial molecules by screening for microbial populations that possess antimicrobial activity against clinically relevant microorganisms.

**Conclusion**

In Nepal, where public health struggles with proper clinical management algorithms, findings from metagenomic next generation sequencing can provide vital information to the clinicians to develop treatment regimens against any infection and to the public health authorities to develop effective management strategies. Thus, metagenomic next generation sequencing is a vital tool for public health management that can investigate known or novel pathogens, outbreaks and complex diseases.

## A11. Understanding the molecular basis for accurate cell division

### Maria Alba Abad, Arulanandam Arockia Jeyaprakash

#### Wellcome Centre for Cell Biology, University of Edinburgh, Edinburgh, EH9 3BF, United Kingdom

##### **Correspondence:** Maria Alba Abad (mabadfe@exseed.ed.ac.uk)

*BMC Proceedings 2023*, **17(Suppl 3):**A11

**Background**

Cell division is an essential biological process important for the development, growth, and repair of all living organisms. During cell division, the genetic information organised in the form of chromosomes, must be equally distributed to the two newly formed daughter cells. Errors in cell division result in cells with an abnormal chromosome number which is often associated with birth defects and diseases like cancer. An extensive network of protein-protein interactions acting at the constricted region of the chromosomes, known as the centromere, is essential to achieve accurate cell division. Our work aims to understand the molecular mechanisms that ensure faithful chromosome segregation by studying the molecular and structural basis of key regulators and associated protein-protein interactions.

**Materials and methods**

We use an interdisciplinary approach that involves the use of protein biochemistry, biophysical and structural techniques combined with cell biology.

**Results**

The Chromosomal Passenger Complex (CPC; composed of Borealin, Survivin, INCENP and Aurora B kinase) is a key regulator of error-free chromosome segregation, and its function is tightly linked to its distinct localisation during cell division. During early stages of cell division, CPC localises at the centromere, where it regulates the attachment of chromosomes to the spindle microtubules and the timing of mitotic progression. We have deciphered the molecular basis for CPC interaction with: 1) nucleosomes, the structural unit of eukaryotic chromosomes, and revealed that this predominantly involves Borealin [1]; and 2) Shugoshin 1 (Sgo1), the protector of cohesion at the centromeric region of chromosomes, mediated via Survivin [2]. Disrupting Borealin-nucleosome or Survivin-Sgo1 interactions led to CPC mis-localisation and caused chromosome segregation defects.

**Conclusion**

Our work establishes that Borealin-nucleosome and Survivin-Sgo1 interactions are essential for proper chromosome association and function of the CPC, and thus for accurate chromosome segregation.

**References**

1. Abad MA, Ruppert JG, Bukuz L, Wear M, Zou J, Webb KM, Kelly DA, Voigt P, Rappsilber J, Earnshaw WC, Jeyaprakash AA. Borealin-nucleosome interaction secures chromosome association of the chromosomal passenger complex. J Cell Biol, 2019, 218 (12): 3912-3925.
2. Abad MA, Gupta T, Hadders MA, Meppelink A, Wopken JP, Blackburn E, Zou J, Gireesh A, Buzuk L, Kelly DA, McHugh T, Rappsilber J, Lens SMA, Jeyaprakash AA. Mechanistic basis for Sgo1-mediated centromere localization and function of the CPC. J Cell Biol, 2022, 221 (8): e202108156.

## A12. Enzyme assay on paper-based analytical devices

### Basant Giri

#### Center for Analytical Sciences, Kathmandu Institute of Applied Sciences, Kathmandu, Nepal

##### **Correspondence:** Basant Giri (bgiri@kias.org.np)

*BMC Proceedings 2023*, **17(Suppl 3):**A12

**Background**

Paper-based analytical devices (PADs) are promising low-cost and easy to use platforms for rapid on-site analysis. The PADs fulfil World Health Organization (WHO)’s ASSURED criteria for point of care (POC) devices. They have attracted increasing attention in the past decade because of their unique advantages.

**Methods**

The PADs are commonly fabricated using Whatman filter or chromatography paper or nitrocellulose paper. Colorimetric detection method is one of the widely used methods employed on PADs assays. Often the colorimetric PADs assays are combined with a smartphone for image acquisition and signal reading, analysis, and reporting of the results. The PADs platforms have been shown to be applicable in biomedical & clinical diagnostics, environmental analysis, food and water quality screening and many others. Enzymes assays have been widely employed in PADs methods for the determination of important target analytes such as creatinine, glucose, cancer biomarkers, human hormones, pesticides, food quality markers etc.

**Conclusion**

In this presentation, I will provide a brief overview of enzyme essays on PADs with examples. In addition, enzyme assays on PADs developed in our lab for the determination of pesticides and milk quality will be explained. One of the challenges of enzyme assays on PADs is the long-term storage of enzymes on the paper substrate at ambient environmental conditions which is essential in POC testing. To address the stability issue, we have developed a sandwich model to retain the activity of enzymes on paper substrates at ambient condition. I will conclude my talk with this sandwich model that helped us retain more than 80% enzyme activity for four months.

## A13. EutM shell protein as building blocks for multifunctional biomaterials

### Anaya Pokhrel^1,2^, Sun-Young Kang^1,2^, Sara Bratsch^1,2^, Claudia Schmidt-Dannert^1,2^

#### ^1^Department of Biochemistry, Molecular Biology & Biophysics, University of Minnesota, Minneapolis, MN, 55455, USA; ^2^BioTechnology Institute, University of Minnesota, St. Paul, MN, 55108, USA

##### **Correspondence:** Claudia Schmidt-Dannert (schmi232@umn.edu)

*BMC Proceedings 2023*, **17(Suppl 3):**A13

**Background**

Bacterial microcompartments (BMCs) are protein-based organelles that encapsulate diverse metabolic pathways inside semipermeable, icosahedral or pseudo icosahedral shells. BMC shell is composed of modular self-assembling proteins, which are valuable targets for bioengineering. EutM shell protein serves as one of the major building blocks for Ethanolamine utilization BMC. EutM assembles as flat hexamers and purified recombinant EutM self-assembles to form 2D-scaffolds. EutM is highly amenable to engineering and tolerates the fusion of diverse N-terminal and C-terminal peptide tags and domains while still self-assembling into robust scaffolds. The EutM shell protein can therefore serve as excellent platform building blocks for the design of diverse functional protein-based nanomaterials.

**Results**

In one recent application, we took advantage of the engineerability and self-assembling properties of this protein for the design of an extracellular protein matrix for the fabrication of a living biocomposite material capable of self-fabrication and regeneration. As a living component of the material, we engineered *Bacillus subtilis* to secrete self-assembling EutM scaffolds for functionalization, and cross-linking of cells. *B. subtilis* was engineered to display SpyTags on polar flagella for cell attachment to SpyCatcher modified secreted EutM scaffolds. A silica biomineralization peptide was genetically fused to EutM to form a silica material with enhanced mechanical properties. We showed that the resulting engineered living material (ELM) can be regenerated from a small piece of the cell containing biocomposite material and that new functions can be readily incorporated by co-cultivation of different engineered *B. subtilis* strains.

**Conclusion**

Our work serves as a framework for the future design of more resilient autonomous self-fabricating ELMs. Applications and prospects for EutM engineering *in vitro* and *in vivo* for synthetic biology and biotechnology will be discussed.

## A14. Overview on genetics and genomics-based research on global health and environmental (biodiversity) studies in Nepal- from Tiger genetics, Aquatic biodiversity assessment using eDNA to viral discovery for determine emerging diseases

### Dibesh Karmacharya

#### ^1^Center for Molecular Dynamics-Nepal (CMDN), BIOVAC Nepal and its consortium of research institutions

##### **Correspondence:** Dibesh Karmacharya (dibesh@cmdn.org)

*BMC Proceedings 2023*, **17(Suppl 3):**A14

**Background**

We conducted several genetics and genomics-based research to understand biodiversity and disease dynamics in Nepal through projects like the Nepal Tiger Genome Project [1], the Nepal Fish Biodiversity Project [2] and the PREDICT Nepal Project [3] (emerging disease research). The Nepal Tiger Genome Project (NTGP), a two-year (June 2011- 2013) research project, built a genetic database of wild Bengal tigers of Nepal. Nepal Fish Biodiversity Project (2016-2019) worked on building fresh water fish genetic databases by using novel environmental DNA (eDNA) based genomic tools. PREDICT was Nepal’s longest running One Health virus surveillance program (2012-2020). This project systematically collected human and animal samples from both rural and urban wildlife interfaces to detect, track, and understand the emergence of new zoonotic pathogens from wildlife and animals (livestock, poultry) that could pose a threat to human health.

**Methods**

With the NTGP, the Center for Molecular Dynamics Nepal (CMDN) built the necessary infrastructure and laboratory capacity to conduct conservation genetics work in Nepal. PCR and sequencing laboratory were set up to conduct Nepal’s first non-invasive based genetics study of the Bengal tigers. With the Nepal Fish Biodiversity project, CMDN built Nepal’s first genomics laboratory with installation of a next generation DNA sequencing machine. CMDN developed field and lab protocols to conduct environmental DNA assessment of river (fish) aquatic biodiversity, thereby profiling fish species from 2 different major river systems. CMDN also developed non-invasive viral discovery molecular techniques to understand emerging diseases in collaboration with the University of California-Davis.

**Results**

Using non-invasive genetics through NTGP, Nepal was able to build the region's first comprehensive Bengal tiger genetic database, giving greater understanding of tiger’s population size, sub-population interactions and gene flow and providing molecular forensic capability to track poached tiger parts by assessing their source of origin. We were able to develop eDNA technology to detect and identify fish species using just water samples; we now have an extensive fish species database of two major river systems of Nepal. This information is now being used to do environmental impact assessment for hydropower development. We conducted seasonal, concurrent sampling of animals and humans in rural and urban communities as well as year-round, hospital-based syndromic sampling in clinical patients of Nepal. Altogether, 26,217 samples were collected from 3,293 animals (bats, birds, rodents and nonhuman primates) and 2,048 humans during eight years of the project. These samples were screened for five viral families—paramyxo, corona-, influenza, filo, and flaviviruses. We were able to detect 119 unique RNA viruses that could potentially cause diseases in humans.

**References**

1. Nepal Tiger Genome Project, 2013, Center for Molecular Dynamics Nepal
2. Nepal Fish Biodiversity Project, 2019, Center for Molecular Dynamics Nepal
3. PREDICT Nepal Project, 2020, Center for Molecular Dynamics Nepal, UC Davis

## A15. Tryptophan-like fluorescence (TLF) for real-time detection in drinking and environmental water samples in Nepal

### Anusa Thapa^1^, Suvechhya Bastola^1,2^, Sishir Gautam^1^, Sanket Bohora^1^, Prashant Waiba^1^, Rijan Maharjan^1^, Prajwal Rajbhandari^2^, Thomas Krauss^3^, Ashim Dhakal^1^

#### ^1^Biophotonics Lab, Phutung Research Institute, Tarakeshor-7, Kathmandu, 44611, Nepal; ^2^Department of Applied Microbiology and Food Technology, Research Institute for Bioscience and Biotechnology, Kathmandu, Nepal; ^3^Department of Physics,University of York, York YO10 5DD, UK

##### **Correspondence:** Anusa Thapa (athapa@pinstitute.org)

*BMC Proceedings 2023*, **17(Suppl 3):**A15

**Background**

A real-time, portable and low-cost detection method for the detection of fecal contamination in drinking water has been in demand, especially in developing countries. Studies [1,2] have demonstrated correlation between the intensity of Tryptophan-like Fluorescence (TLF, Excitation/Emission = 280 nm / 350 nm) from environmental water samples to the number of colony forming units (CFUs) of TTCs (thermotolerant coliforms) present in the water sample. In this work, we validate that TLF-fluorometry is applicable for detection of fecal contamination for the range of water samples collected from various important sources in the Kathmandu valley by analyzing their microbiological and UV-fluorescence properties.

**Material and methods**

We collected 157 randomized water samples from the Kathmandu valley. Samples from the river water (58), underground water (24), refill jar water (21), mineral bottled water (25), lab-grade water (6) and municipal-supplied household water (23) were tested for their respective TLF (ppb) and number of TTC (CFU/100mL).

**Results**

We were able to classify the corresponding range of TLF in the WHO risk level for fecal contamination, TTCs (CFU/100ml). For the respective low-risk, intermediate, high and very high-risk WHO risk categories, we observed the mean TLF (ppb) of 2.9, 9.6, 12.3 and 155.4 with corresponding standard deviations 4.2, 13.8, 20.0 and 212.5.

**Conclusion**

We provide the range of TLF measurements for classifying water samples from Kathmandu Valley according to WHO risk categories with a confidence level of 68% .

**Acknowledgement**

We would like to thank Nepal Academy of Science and Technology (NAST), Biotechnology Department, for providing us with Milli Q lab grade water. This work was supported by EPSRC’s GCRF Grant # EP/T020008/1 & EP/W524165/1.

**References**

1. Cumberland S, Bridgeman J, Baker A, Sterling M, Ward D. Fluorescence spectroscopy as a tool for determining microbial quality in potable water applications. Environmental technology. 2012 Mar 1;33(6):687-93.
2. Sorensen JP, Baker A, Cumberland SA, Lapworth DJ, MacDonald AM, Pedley S, Taylor RG, Ward JS. Real-time detection of faecally contaminated drinking water with tryptophan-like fluorescence: defining threshold values. Science of the Total Environment. 2018 May 1; 622:1250-7

## A16. Antibiotic resistance surveillance and molecular characterization of ESBL and Carbapenemase genes present in bacteria isolated from fruits and vegetables sold in Kathmandu

### Jenny Shah^1,2^, Ashish Bhusal^1,2^, Bishnu Marasini^2^, Era Tuladhar^2^, Mitesh Shrestha^1^

#### ^1^Department of Applied Microbiology and Food Technology, Research Institute for Bioscience and Biotechnology, Kathmandu, Nepal; ^2^National College, Khusibu, Kathmandu, Nepal

##### **Correspondence:** Mitesh Shrestha (mitesh.shrestha@ribb.org.np)

*BMC Proceedings 2023*, **17(Suppl 3):**A16

**Background**

Antibiotic resistance has become a global threat. Resistant bacteria can spread between individuals, animals, and the environment. Foods can serve as a vehicle for transmitting antibiotic resistant bacteria, especially the fresh fruits and vegetables which are often eaten raw can become a source for food borne illness.

**Materials and methods**

A total of 40 samples including fresh fruits and vegetables were collected from vendors around ten major hospitals and seven major market places in Kathmandu. Samples were transported in sterile condition to the laboratory where they were washed with MRD (Maximum Recovery Diluent) separately, followed by 3 hrs. incubation. Bacteria were then isolated by using spread plate technique in MacConkey and VRBG (violet red bile glucose) agar. Gram staining followed by different biochemical tests were performed for the preliminary identification of bacteria. Antimicrobial susceptibility test was done using twenty different antibiotic discs by disk diffusion method. Polymerase chain reaction was carried out for the detection of ESBL and Carbapenem genes.

**Results**

Of the 162 isolates from different samples, 60 (37%) were identified as ESBL producers and 7 (4%) were identified as carbapenemase producers. Among these, blaTEM (19), blaSHV(2), blaOXA(2), blaIMP(1) and blaNDM(1) were found.

**Conclusion**

In conclusion, fresh produce and fruits could be one of the potential sources for transmission of antibiotic resistant bacterial pathogens.

**Acknowledgement**

The study was supported by the International Foundation for Science (IFS).

## A17. Nanophotonic waveguide integrated evanescent upconversion spectroscopy for biomedical applications

### Ankit Poudel, Pravin Bhattarai, Rijan Maharjan, Ashim Dhakal

#### ^1^Phutung Research Institute, Kathmandu, Nepal

##### **Correspondence:** Ashim Dhakal (ad@pinstitute.org)

*BMC Proceedings 2023*, **17(Suppl 3):**A17

**Background**

Enormous progress has been made in demonstrating various applications of nanophotonics. This emerging application of PICs demands a broad optical wavelength range. SiN waveguide system has proven to be the prominent candidate in that regard [1]. Furthermore, nanophotonic waveguide enhanced Raman spectroscopy has recently been demonstrated [2]. Nanophotonic waveguides can be used to evanescently excite and collect upconverted signals. Fascination of upconversion in Rubrene molecules [3] at near infrared (NIR) wavelengths makes it an ideal choice for analysis.

**Devices and methods**

We designed nanophotonic circuits optimized for SiN as core material and SiO_2_ as substrate with refractive index(n) 2.05 and 1.45 respectively, keeping the upper surface open. We conducted a modal analysis using the Finite Difference Eigenmode (FDE) solver for optimization.

**Theoretical analysis**

In nanophotonic waveguides, confinement of light in a sub-nanoscale enhances the spontaneous emission rate of particles which are in close proximity, suggested by E. M. Purcell in 1946 [4]. Using this as a fundamental principle we calculate the specific conversion efficiency μ_0_ of the waveguide.

**Results**

The specific conversion efficiency (μ_0_) calculated on top of the cladding with a function of increasing width of the waveguide. μ_0_ decreases with the increasing width of the waveguide which corresponds to the increasing model confinement of the field in the core region thereby decreasing the evanescent field interaction with the analyte. Also, the TM polarized mode generally performs better than the TE polarized mode leading to a higher evanescent interaction with the analyte.

**Conclusion**

Here we have devised a theoretical and experimental model for the evanescent upconversion in SiN nanophotonic waveguides. We used FDTD simulation interface to achieve accurate simulation of SiNOI circuit passive components. We used a cross section of 300 x 520 nm^2^ SiN waveguide for λ_0_ = 800 nm. We anticipate that this type of device can add new functionality to the silicon photonics ’tool-kit’ for various biosensing applications.

**References**

1. Blumenthal J. D, Heideman R, Geuzebroek D, Leinse A and Roeloffzen C. Silicon Nitride in Silicon Photonics, in Proceedings of the IEEE, 106, no. 12, pp. 2209-2231, Dec. 2018, doi: 10.1109/JPROC.2018.2861576.
2. Dhakal A. Nanophotonic Waveguide Enhanced Raman Spectroscopy, Ghent University. Ghent, 2015-2016.
3. Cheng Y. Y, Khoury T, Clady G. R, Tayebjee M. J, Ekins-Daukes J. N, Crossley M. J, & Schmidt T. W. On the efficiency limit of triplet–triplet annihilation for photochemical upconversion, Physical Chemistry Chemical Physics, 12(1), 66-71, 2010.
4. Purcell M. E. Proceedings of the American Physical Society: Spontaneous Emission Probabilities at Radio Frequencies, Physical Review. American Physical Society (APS), 1946.

## A18. Extract of *C. parqui* reduces the proliferation of triple negative breast cancer cells MDA-MB-231 *in vitro*

### Asbin B. Chand^1^, Pragati Pradhan^1^, Deena Shrestha^2^, Bijay Bajracharya^2^, Rajani Malla^1^, Roshan Lal Shrestha^2^, Jivan Shakya^3^

#### ^1^Central Department of Biotechnology, Tribhuvan University, Kirtipur, Nepal; ^2^Centre for Health and Disease Studies, Kathmandu, Nepal; ^3^Central Department of Microbiology, Tribhuvan University, Kirtipur, Nepal

##### **Correspondence:** Asbin B. Chand (asbinchand99@gmail.com)

*BMC Proceedings 2023*, **17(Suppl 3):**A18

**Background**

There has been a constant demand for targeted therapies against cancers as cancer represents a global burden to both the health system and economy [1]. Most clinically used anticancer drugs trigger cytotoxicity through apoptosis, DNA damage, genome instability and mitotic catastrophe. Nepal’s diverse ecological landscape is favorable for many medicinal plants rich in a diverse range of compounds that may have anti-cancer effects. Thus, we screened selected medicinal plants of Nepal for their anticancer efficacy.

**Materials and methods**

Plant samples were collected from various regions of Nepal. Crude methanolic extracts were prepared from dried leaf parts and vacuum evaporated for storage until further experiments. Qualitative phytochemical screening followed by quantitative estimation of total flavonoid content (TFC), total phenolic content (TPC) and antioxidant property were carried out using standard protocols. Cell proliferation inhibition assay was used for cytotoxicity assay and Annexin V positive cells were sorted using FACSCalibur. Triple Negative Breast Cancer (TNBC) cell line, MDA-MB-231 was used as a cell line modal.

**Results**

Quantitative estimation yielded the highest concentration of TPC in *Eucalyptus alba* bark extract (422.37 ±9.34 mgGAE/gm crude extract). Similarly, quantitative estimation of TFC yielded its highest concentration in *Ageratina adenophora* (156.94±0.7 mgQE/g crude extract). Anti-inflammatory potential was assessed by DPPH free radical scavenging activity and showed that *Eucalyptus alba* bark extracts had IC_50_ value as low as 0.046 mg/mL. However, few plant extracts such as *Solanum nigrum* and *Cestrum parqui (C.parqui)* did not show DPPH radical scavenging activity. While treatment with *C. parqui* extract inhibited proliferation of MDA-MB-231 cells by more than 50%, other plant extracts had null effects on cell proliferation. Interestingly, treatment with extract from *Tinospora cordifolia* showed enhanced cell proliferation. In accordance with the cell proliferation inhibition results, *C. parqui* induced the highest percentage of cell death via apoptosis (68.7%) at 24 hrs which upon prolonged co-incubation proceeded to necrosis. Similarly, *E. alba* leaf extract also induced apoptosis in approx. 61.7% of the MDA-MB-231 cells during 24hrs co-incubation which proceeded to necrosis upon prolonged co-incubation.

**Conclusions**

We conclude that high flavonoid compounds do not correlate with reduced cell proliferation in TNBC cells. *C. parqui* and *E. alba* leaf extracts were found to inhibit cell proliferation significantly. Surprisingly, this study demonstrated that the *Tinospora cordifolia* methanolic extract stimulates cell proliferation by more than 40%. Currently, we are investigating the active compound responsible for inducing cell death in *C. parqui* extract.

**Acknowledgement**

The work was supported by The World Academy of Sciences, Grant no. 19-281 RG/BIO/AS_G.

**References**

1. Bray, F., Ferlay, J., Soerjomataram, I., Siegel, R. L., Torre, L. A., & Jemal, A. (2018). Global cancer statistics 2018: GLOBOCAN estimates of incidence and mortality worldwide for 36 cancers in 185 countries. *CA: A Cancer Journal for Clinicians*, *68*(6), 394–424. 10.3322/caac.21492**.**

## A19. In-vitro insecticidal activities of native *Bacillus thuringiensis* combinations against the tomato leaf miner, *Tuta absoluta*

### Bibechana Dhital^1,2^, Kushal Thapa^1,2^, Bishnu Marasini^2^, Era Tuladhar^2^, Mitesh Shrestha^1^

#### ^1^Department of Applied Microbiology and Food Technology, Research Institute for Bioscience and Biotechnology, Kathmandu, Nepal; ^2^National College, Kathmandu, Nepal

##### **Correspondence:** Mitesh Shrestha (mitesh.shrestha@ribb.org.np)

*BMC Proceedings 2023*, **17(Suppl 3):**A19

**Background**

*Bacillus thuringiensis* (Bt) is one of the most used microbial control agents for commercially important agricultural insect pests owing to its highly efficient insecticidal property and safety in use. Cry proteins are one of the primary proteins responsible for its insecticidal properties. The purpose of this study was to analyse insecticidal activities of native Bt against common insect pests like tomato leaf miners (*Tuta absoluta*).

**Materials and methods**

To this end, one hundred and forty-four soil samples were collected from various parts of Nepal for Bt isolation. From these, 12 isolates were confirmed as Bt based on their colony characteristics, gram staining, endospore staining, observation of crystal proteins when stained with amido black and 16s rRNA sequencing. Crystal protein diversity was observed through SDS-PAGE. Compatibility assay was carried out to develop combinations of bacteria that would be used for insecticidal assay. In this way, four (three non-redundant and one with repeated bacteria) combinations were made, each containing four bacterial isolates.

**Results**

Varied results were obtained for each of the combinations for insecticidal assay, with highest (80 %) mortality being observed for Combination 1.

**Conclusions**

Hence, we can conclude that the combination of different strains of Bt had a synergistic effect on insect mortality, which could help us to address the rising resistance of the insects against the most preferred single bacteria method.

**Acknowledgement**

The study was supported by Applied Microbiology International (formerly Society for Applied Microbiology).

## A20. Phytochemical assessment by GC-MS and pharmacological potentials: comparative study of *Allium hypsistum* and *Allium przewalskanium*

### Deepak Kumar Shrestha, Ajay Singh

#### Department of Chemistry, School of Applied & Life Sciences, Uttaranchal University, Dehradun, Uttarakhand, India-248007

##### **Correspondence:** Deepak Kumar Shrestha (shresdeepak@gmail.com)

*BMC Proceedings 2023*, **17(Suppl 3):**A20

**Background**

*Allium hypsistum* (common name-jimbu) and *Allium przewalskanium* (common name-lamboo) are popular herbs which are commonly used spices and traditional medicines in high altitude area of Nepal, India and China; however, chemical profiling and experimental therapeutic evidence are still unsettled [1]. This study is focused to assess chemical constituents and selected pharmacological properties- antidiabetic, lipid lowering and antioxidant activities of ethanolic extracts of *Allium hypsistum* (EEAH*)* and *Allium przewalskanium* (EEAP).

**Materials and methods**

The aerial parts of the plants collected shade dried for 15 to 21 days at RT. The extracts were prepared from soxhlet extraction method and their GC-MS analysis was carried out using Agilent 7890A GC-Agilent 5975C inert MSD with triple axis detector [2]. Antidiabetic and lipid lowering activities were evaluated by daily exposure of 500mg/kg body weight of the extracts in streptozotocin (STZ) induced diabetic albino mice. The fasting blood glucose level (BGL) was measured after 72hrs of STZ administration (baseline) and, at 7th day and 14th day from the baseline. The lipid profiling (total cholesterol (TC), triglycerides (TG), high density lipoprotein (HDL), low density lipoprotein (LDL) and very low-density lipoprotein (VLDL)) was estimated at 14th day [3, 4]. Further, *in vitro* antioxidant activities of the extracts were measured by their TPC (Folin-Ciocalteu assay) and % RSA (DPHH scavenging assay) [5].

**Results**

GC-MS analysis of EEAH detected six different compounds; phenol- 2,4-bis (1,1-dimethylethyl), 2-fluoro-5- (trifluoromethyl) acetophenone, 2,4,5,5, 8a-Pentamethyl-6,7,8,8a-tetrahydro-5H-chromene, didodecyl phthalate, phthalic acid- ethyl pentyl ester and phthalic acid-isopropyl propyl ester. Simultaneously, three compounds were identified in EEAP, they are; methyl salicylate, toluene and cylclobutene-2-propenylidene. Some of these compounds were already illustrated with their pharmacological activities in previous study [6], however, 2-fluoro-5- (trifluoromethyl) acetophenone, didodecyl phthalate, phthalic acid-isopropyl propyl ester and cylclobutene-2-propenylidene are not well studied for their pharmacological applications. In both EEAH and EEAP, there were significantly decreased (*p*<0.01) in blood glucose level (BGL) of diabetic induced mice at 7^th^ and 14^th^ day exposure. On the 7th day, EEAH showed 37.04% reduction whereas EEAP showed 26.92% reduction in BGL. Similarly, on the 14th day, 51.41% reduction by EEAH and 36.70% reduction by EEAP were observed in BGL. Moreover, exposure of EEAH and EEAP to the diabetic mice till 14^th^ day, serum lipid profiles (TC, TG, HDL, LDL and VLDL) were found to be improved while comparing with the control groups of mice (diabetic control and normal control). *In vitro* antioxidants, TPC were found to be 172±9.23 mgGAE/100mg in EEAH which was significantly higher (*p*<0.01) than EEAP (122±1.15 mgGAE/100mg). Similarly, DPPH activity of EEAH (59.44±1.27%RSA) was found significantly higher ((*p*<0.01) than EEAP (39.53±0.42).

**Conclusion**

From GC-MS analysis, some promising chemical constituents with the pharmacological activities were identified in EEAH and EEAP; however, some of them are still unknown for their pharmacological roles. Both extracts showed significant reduction in BGL and improvement of lipid profile in diabetic induced mice. Similarly, the extracts exhibited *in vitro* antioxidant activities. Interestingly, EEAH showed significantly higher pharmacological potentials than EEAP. Hence, both EEAH and EEAP were found to be promising sources for phytomedicine with their pharmacological activities.

**References**

1. Nepal, R.c., Status, Use and Management of Jimbu (Allium spp.):A Case Study from Upper Mustang, Nepal. 2006
2. Olivia, N.U., U.C. Goodness, and O.M. Obinna, Phytochemical profiling and GC-MS analysis of aqueous methanol fraction of Hibiscus asper leaves. Future Journal of Pharmaceutical Sciences, 2021. 7(1): p. 59.
3. Srivastava, A.K., A. Mukerjee, and A. Tripathi, Antidiabetic and antihyperlipidemic activities of Cucumis melo var. momordica fruit extract on experimental animals. Future Journal of Pharmaceutical Sciences, 2020. 6(1): p. 92.
4. Pochhi, M., Evaluation of Antidiabetic potential and Hypolipidemic activity of Coccinia indica (leaves) in Diabetic Albino rats. Asian Journal of Medical Sciences, 2019. 10(4): p. 49-54.
5. Chernukha, I., et al., Antioxidant effect of ethanolic onion (Allium cepa) husk extract in ageing rats. Saudi Journal of Biological Sciences, 2021. 28(5): p. 2877-2885.
6. Miri, S.M. and A. Roughani. Allium species growing in Iran: Chemical compositions and pharmacological activity. in Proceedings of the First National Congress and International Fair of Medicinal Plants and Strategies for Persian Medicine that Affect Diabetes, October. 2018.

## A21. DNA barcoding of coffee varieties grown at coffee development center, Gulmi, Nepal using ITS2 gene sequence

### Shreejan Pokharel^1^, Samsher Basnet^2^, Anish Basnet^3^, Ashmita Mainali^3^, Sadikshya Rijal^3^, Asmita Shrestha^3^, Gyanu Raj Pandey^3^

#### ^1^National Biotechnology Research Center, National Agricultural Research Council, Lalitpur, Nepal; ^2^National Entomology Research Center, National Agricultural Research Council, Lalitpur, Nepal; ^3^Shubham Biotech Nepal Pvt. Ltd., Bharatpur-29, Chitwan, Nepal

##### **Correspondence:** Gyanu Raj Pandey (gyanupandey9@gmail.com)

*BMC Proceedings 2023*, **17(Suppl 3):**A21

**Background**

Nepali coffee is labeled as specialty coffee in several foreign markets and is noted for its organic certification and fair-trade practices [1]. Despite the long history of coffee plantations in Nepal, the taxonomic study has not been conducted to date, and genetic diversity is unknown. Instead of the conventional method of identification based on morphological characteristics, the most efficient method which can be used for species identification is the molecular method involving a short sequence known as barcode. ITS (internal transcribed spacer) region of nuclear ribosomal cistron shows a high level of intra-specific variability and thus, is often used for intra-specific discrimination in angiosperms [2].

**Methods**

Young coffee leaves samples (n=25) were collected from the Coffee Development Centre (CDC) and stored in poly bags at -80 °C for DNA extraction. DNA was extracted using Doyle and Doyle method [3] with some optimizations. Extracted DNA was evaluated by using 0.8% agarose gel electrophoresis at 70 V for 1 hr. ITS region of extracted DNA was amplified by using PCR and sequencing was carried out using a sequencing primer. Pairwise comparisons of obtained sequences were performed using the maximum likelihood method model and analyzed in MEGA X.

**Results**

The BLASTN analysis showed that the coffee samples (n=25) had 100% similarity to *C. Arabica.* There were three major cultivars of *C. Arabica*; Typica, Bourbon, and near to Java and Typica.

**Conclusions**

In this study, Maximum-likelihood phylogenetic Tree generated from ITS sequence data has been successful in discriminating the coffee species. We recommend researchers to use ITS as a phylogeny reconstruction tool for intra-species discrimination in coffee samples.

**Acknowledgment**

The authors thankfully acknowledge Coffee Development Center for providing the research funding and Shubham Biotech Nepal Pvt. Ltd.for supporting with research facilities and space.

**References**

1. National Tea and Coffee Development Board: Government of Nepal. (2022). [https://www.teacoffee.gov.np/]
2. Kress, W., Wurdack, K., Zimmer, E., Weigt, L., & Janzen, D. (2005). Use of DNA barcodes to identify flowering plants. *Proceedings Of The National Academy Of Sciences*, *102*(23), 8369-8374. doi: 10.1073/pnas.0503123102.
3. J. J. Doyle and J. L. Doyle, “Doyle_plantDNAextractCTAB_1987.pdf,” Phytochemical Bulletin, vol. 19, no. 1. pp. 11–15, 1987.

## A22. Valorization of whey-rich dairy wastewater by microalgae cultivation

### Basanta K Chaudhary^1,2^, Bibek C Mahaseth^1,2^, Kamana Dawadi^1,2^, Lochan Pandeya^1,2^, Nasla Shakya^1^, Sanjaya Lama^1^

#### ^1^Department of Plant Physiology and Environmental Sciences, Research Institute for Bioscience and Biotechnology, Kathmandu, Nepal; ^2^Kantipur Valley College, Kumaripati, Lalitpur, Nepal

##### **Correspondence:** Sanjaya Lama (moktansanjaya@gmail.com)

*BMC Proceedings 2023*, **17(Suppl 3):**A22

**Background**

Microalgae are highly diverse, rapidly proliferating, photosynthetic, aquatic microbes with cell biomass rich in carbohydrates, protein, lipids and various other bioactive molecules of commercial interest [1, 2]. The primary microalgae-based biorefinery process includes; species-strain selection, cultivation, harvesting, and processing into targeted products [1, 3]. The cultivation stage significantly contributes to the total cost of biorefinery [4]. The growth medium is one of the crucial cultivation parameters that can be optimized to reduce production costs [2]. One of the emerging studies to achieve the sustainability of large-scale production involve nutrient recovery from the waste resources such as nutrient-loaded wastewater [5].

**Results**

This study investigated the growth of microalga *Chlorella* sp. (obtained from RIBB culture collection) in cheese whey compared to standard Bold’s Basal Medium (BBM). The cheese whey was obtained from the local dairy farm, which is generally discarded or underutilized as a waste by-product. The optimum growth (based on cell counting and total chlorophyll content) analogous to BBM was observed in 20 % (v/v) diluted cheese whey (CW). The dry cell weights of 0.80 g/L and 0.69 g/L were obtained for CW and BBM on the 12^th^ day of culture. The growth characteristics in CW were replicated in scale-up (18 L) batch cultivation. The *Chlorella* sp. biomass from the scale-up culture was harvested and dried to obtain a fine-grained dry powder. The dry biomass was supplemented (1 % and 2 % (w/v)) in the MRS media used for the culture of *Lactobacillus* sp. (obtained from RIBB culture collection). The supplementation of *Chlorella* sp. dry biomass (2 % (w/v)) enhanced the growth of the *Lactobacillus* sp. A higher growth rate (0.09 hr^-1^) and shorter doubling time (8.15 hr) were observed in *Chlorella-*supplemented MRS compared to MRS alone (0.07 hr^-1^ and 10.57 hr). Additionally, high β-galactosidase activity was detected in the dry *Chlorella*-supplemented *Lactobacillus* culture. Thus, this study explored the potential of resource recovery from the dairy waste stream for the microalgal cultivation and application of the biomass to enhance the growth of industrially important probiotic species such as *Lactobacillus*.

**References**

1. Foley PM, Beach ES, Zimmerman JB. Algae as a source of renewable chemicals: opportunities and challenges. Green Chemistry. 2011;13(6):1399-405.
2. Rizwan M, Mujtaba G, Memon SA, Lee K, Rashid N. Exploring the potential of microalgae for new biotechnology applications and beyond: A review. Renewable and Sustainable Energy Reviews. 2018; 92:394-404.
3. Gifuni I, Pollio A, Safi C, Marzocchella A, Olivieri G. Current Bottlenecks and Challenges of the Microalgal Biorefinery. Trends in Biotechnology. 2019;37(3):242-52.
4. Daneshvar E, Sik Ok Y, Tavakoli S, Sarkar B, Shaheen SM, Hong H, et al. Insights into upstream processing of microalgae: A review. Bioresource Technology. 2021; 329:124870.
5. Li K, Liu Q, Fang F, Luo R, Lu Q, Zhou W, et al. Microalgae-based wastewater treatment for nutrients recovery: A review. Bioresource Technology. 2019;291:121934.

## A23. *Bacillus cereus* in ready-to-eat foods available in Kathmandu

### Charu Arjyal^1^, Sagarika Manandhar^2^

#### ^1^Department of Microbiology, Padma Kanya Multiple Campus, Tribhuvan University, Kirtipur, Nepal; ^2^Department of Microbiology, Tri-Chandra Multiple Campus, Tribhuvan University, Kirtipur, Nepal

##### **Correspondence:** Charu Arjyal (carjyal@gmail.com)

*BMC Proceedings 2023*, **17(Suppl 3):**A23

**Background**

The food items that do not need to be prepared significantly except reheating or completing the cooking procedure are referred to as ready-to-eat foods [1]. Like other food items, ready-to-eat food items can also harbor microorganisms and can cause the transmission of foodborne microorganisms into the human body, if not handled and stored properly. *Bacillus cereus* is more known to cause ‘fried rice syndrome’, along with different types of food poisoning caused by the emetic toxin or enterotoxin producing microorganism [2]. The aim of this study was to assess the ready-to-eat food samples available in Kathmandu and detect if *Bacillus cereus* were present.

**Materials and methods**

The study period was from February 2021 to January 2022 during which the convenient sampling method was employed to collect 240 ready-to-eat food samples from different food outlets in Kathmandu. The food samples were grouped into three main categories- rice dishes (plain rice, fried rice, biryani), bakery items (pizza base, patties, and other bread items), and dairy items (cheese, cream, yogurt). The samples were processed following the standard microbiological procedures and the bacterial isolates were identified as *Bacillus cereus* with the help of different biochemical tests.

**Results**

Out of 240 food samples (80 each from the three categories) analyzed, 40% of the samples showed the presence of *Bacillus cereus* and was most prevalent in bakery items followed by rice dishes and dairy items (50%, 42.5%, and 28% of the samples tested showed the presence of *Bacillus cereus* respectively). One of the *Bacillus cereus* isolates was an emetic toxin-producing type as detected from the phenotypic analysis. The contamination of *Bacillus cereus* ranged from 1.64 log cfu/g (colony forming units/gram) to 6.83 log cfu/g and 20.8% of ready-to-eat food samples were found to be potentially hazardous (>5 log cfu/g), because of the potential toxin-producing ability of the contaminant bacteria.

**Conclusions**

It is therefore imperative to maintain appropriate standards during the preparation, packaging, storage, and transport and regular monitoring of ready-to-eat food items to prevent any food poisoning incidents.

**Acknowledgements**

This research was funded by the University Grants Commission, Nepal as part of a Small RDI (Research, Development and Innovation) Grant, 2076-77, SRDIG-76/77-S&T-4. The authors are grateful to the Departments of Microbiology, Padma Kanya Multiple Campus and Tri-Chandra Multiple Campus and to Mr. Saroj Paudel of Nepalese Farming Institute, Kathmandu.

**References**

1. Food Safety Authority of Ireland. Guidelines for the interpretation of results of microbiological testing of ready-to-eat foods placed in the market; 2020. 3p. ISBN: 0-9539183-5-1
2. Ross, R. Live Science [Internet]. New York: Future US, Inc.; 2019 [Updated 2019 May 1; cited 2021 May 26]. Available from https://www.livescience.com/65374-bacillus-cereus-fried-rice-syndrome.html

## A24. Co-circulation of *Orientia tsutsugamushi*, *Anaplasma*, and *Leptospira* bacteria among febrile patients in southern Nepal

### Adesh Baral^1^ , Prakriti Karki^1^ , Minu Singh^1^, Binod Rayamajhee^1^, Pradeep Oli^2^, Anurag Adhikari^1^

#### ^1^Kathmandu Research Institute for Biological Sciences, Lalitpur, Nepal; ^2^Sagarmatha Diagnostic and Polyclinic, Nepalgunj, Bheri

##### **Correspondence:** Anurag Adhikari (adhikari.a@kribs.org.np)

*BMC Proceedings 2023*, **17(Suppl 3):**A24

**Background**

In Nepal, febrile illness is one of the most common reasons for seeking medical attention. However, the limited capacity of diagnostic and microbiological laboratory facilities thus results in many unreported cases of fever with unknown origin. Among the reported febrile cases, the Malaria, Dengue and Salmonella are among the top diagnosis, whereas recently the tick-borne pathogens including the Rickettsia spp. (typhus) has been attributed to a significant fraction of such diagnoses in Nepal. This study aims to utilize a polymerase chain reaction-based assay to identify the circulating tick-borne pathogens among febrile patients from South-West Nepal.

**Materials and methods**

This study utilized Malaria, Dengue, and Salmonella negative acute febrile patient’s whole venous blood to extract, amplify and partially sequence the 47kDa and 56kDa region of *Orientia tsutsugamushi*, groEL gene of *Anaplasma*, and rpoB gene of *Leptospira* to identify the contemporary phylogenomic of these tick-borne bacteria. Then, the phylogenetic tree construction and analysis of the sequenced genes were done using Mega-X software.

**Results**

Among thirty-six febrile participants in the study, 16 were positive for *O. tsutsugamushi* 47kDa in-house PCR, however, only 14 were positive for *O. tsutsugamushi* 56kDa in-house PCR. Among thirty-six suspected patients for typhus, all patients’ samples 36/36 were positive for Scrub Typhus Detect^TM^ IgM Rapid Test. Among the total febrile patients, two patients tested positive for *Anaplasma sp.* groEL gene PCR (2/36), and also two of those patients were positive for *Leptospira sp.* rpoB gene PCR. The phylogenetic analysis of the partial genome of *O. tsutsugamushi* 47kDa gene (GenBank accession: OL770337-OL770352) showed a close relation with Karp-UK strain (14/16), as well with CRF93-Thailand strain (1/16), and Karp-Thailand strain (1/16). Similarly, phylogenetic analysis of *O. tsutsugamushi* 56kDa gene (GenBank accession: OL770323-OL770336) showed close relation with Gilliam-Bangladesh strain (5/14), Karp-Bangladesh strain (4/14), Gilliam-UK strain (2/14), Shimokoshi-Taiwan strain (2/14), and with Vietnam strain (1/14). We also show that the patient derived groEL gene of *Anaplasma* (GenBank accession: OL770355 -OL770356) was closely related to D-GB-gro-8-South Korea strain (2/2), and rpoB gene of *Leptospira* (GenBank accession: OL770353-OL770354) was closely related to Linhai 56609-China strain (2/2 of the patients).

**Conclusions**

This study shows for the first time that the Karp, Gilliam, and Shimokoshi strains of *Orientia tsutsugamushi* are circulating in southern Nepal. Additionally, this is the first study to identify the co-presence of *Anaplasma*, and *Leptospira* in the febrile symptomatic patient. Further genotype and serotype screening study for these bacteria among febrile patients is a current need so as to identify the tick-borne bacterial disease burden among Nepalese population.

## A25. Adsorption of Dimethylarsinic acid (DMA) from aqueous solution on iron aluminum-based adsorbents

### Naina Byanjankar, Tista Prasai Joshi

#### Environment and Climate Study Laboratory, Faculty of Science, Nepal Academy of Science and Technology, Khumaltar, Lalitpur, Nepal

##### **Correspondence:** Naina Byanjankar (benznaina@gmail.com)

*BMC Proceedings 2023*, **17(Suppl 3):**A25

**Background**

The involvement of anthropogenic activities including mining and application of pesticides in agricultural fields has been a contributing factor for increasing the concentration of methylated arsenic in water sources, posing a serious threat to the environment and human health. Therefore, removal of methylated arsenic from water applying an efficient method is required. Treatment of water using adsorption techniques has been mostly used being cost-effective, the release of the low amount of residues, easy operation and regeneration capability. The study was carried out with the objective of preparing an iron aluminum-based adsorbent for the adsorption of dimethylarsinic acid from an aqueous solution.

**Materials and methods**

The adsorbent prepared were characterized using X-Ray Diffraction (XRD), zeta potential, and particle size to determine their structural properties. The adsorption capacity of the prepared adsorbent was determined by application of adsorption kinetics, adsorption isotherm, and effect of pH which was compared with single metal oxide alumina.

**Results**

The XRD pattern of Fe-Al showed few crystalline peaks indicating the adsorbent to be slightly crystalline whereas alumina was amorphous in nature. The results of the adsorption isotherm showed the adsorption capacity of Fe-Al and alumina increased with the increasing concentration of DMA. The adsorption capacity of Fe-Al and alumina was found to be 2.44 mg/g and 1.33 mg/g respectively. The adsorption of DMA increased rapidly in the initial stage and slowed down with increasing time periods. Adsorption of DMA onto Fe-Al and alumina was more favorable at pH 5.0 and pH 6.0 respectively. The interaction characteristics of DMA and adsorbents were analyzed by applying FTIR analysis.

**Conclusion**

Fe-Al showed higher removal capacity than alumina thus Fe-AL adsorbent could be effective for the removal of DMA from aqueous solution.

## A26. Low-cost Polymerase Chain Reaction (PCR) technology for the detection of DNA amplicons

### Prashant Waiba^1^, Pravin Bhattarai^1^, Mitesh Shrestha^2^, Ashim Dhakal^1^

#### ^1^Phutung Research Institute, Kathmandu, Nepal; ^2^Department of Applied Microbiology and Food Technology, Research Institute for Bioscience and Biotechnology, Kathmandu, Nepal

##### **Correspondence:** Ashim Dhakal (ad@pinstitute.org)

*BMC Proceedings 2023*, **17(Suppl 3):**A26

**Background**

The design of Pagoda PCR is gleaned from the OpenPCR concept. The reliability of PCR results is dependent on how precisely the intended amplicons are amplified during the thermocycling process [1]. In Pagoda PCR, a single *Peltier module* is used with a commonly available *CPU cooler fan* to heat and cool the thermal block during heating and cooling cycles while, and a separate *heating strip* on the lid ramps up the temperature to 200°C. A simple desktop interface is developed using html and linked to *ESP8266 WIFI module* through Arduino IDE, so that any computer connected to the device defined IP address can access the console which enables the user manually set the PCR protocols.

**Materials and methods**

Key systems driving the temperature regulation include a Hebei TEC1-12710HTS Peltier module, resistive heating element, and a cooling fan from a commercial PC. The temperature control system of the device includes an aluminum thermal block which was heated and cooled with a PID loop [2], with instant temperature measurement performed with an NTC thermistor. The temperature is also controlled efficiently by the lid which prevents the water condensation on the lid, and evaporation of samples.

**Results and discussion**

A test of four samples was run for ITS1/4 gene amplification. The protocol in the machine was set to Initial Denaturation at 95°C for 2 min followed by 35 cycles of Denaturation at 95°C /30 sec; Annealing at 56°C/50 sec, and Extension at 73 °C/140 sec. Then, a final extension was performed for 5 minutes at 73°C, ending with a 4°C hold. After the thermocycling, the amplified product was separated in Agarose Gel Electrophoresis (1%) along with a reference ladder DNA (N3231S). An amplicon of size 700bp was visualized under UV transilluminator which validated the reliability of PagodaPCR performance as a thermocycler.

**Acknowledgements**

Nepal Academy of Science and Technology, Research Institute for Bioscience and Biotechnology, NinjaPCR, Pawan Bioscientific, and Mr. Suman Basnet.

**References**

1. WEIER HEINZULRICH, GRAY JOEW. A programmable system to perform the polymerase chain reaction. DNA. 1988;7(6):441–7.
2. Kiam Heong Ang, Chong G, Yun Li. PID control system analysis, design, and Technology. IEEE Transactions on Control Systems Technology. 2005;13(4):559–76.

## A27. Degradation of potato peels using amylase and pectinase producing fungal strain in electrochemical cell and byproduct analysis

### Puja Bhatt, Jarina Joshi

#### Central Department of Biotechnology, Tribhuvan University, Kirtipur, Nepal

##### **Correspondence:** Jarina Joshi (jarinarjoshi@gmail.com)

*BMC Proceedings 2023*, **17(Suppl 3):**A27

**Background**

Potato peels are the most abundant waste in Asian households. Degradation of the waste using amylase and pectinase producing fungal strain in Microbial fuel cell (MFC) can be a sustainable and economic strategy for solid waste management and hence alternative electricity generation [1]. Microorganisms or enzymes can be utilized to generate electricity in microbial fuel cells (MFCs), which has been known for several years [2].

**Materials and methods**

Fungal strains were isolated from soil and then tested for production of amylase and pectinase enzymes. Potato peel sample was processed and physical characterization was done. Microbial fuel cell was constructed using a finely pasted potato peel waste sample in anode and acetate buffer in cathode. MFC operation was done using different concentration of sample in anode and various catholytes in cathode for enhancement of electricity production. The electrode was modified by coating of the graphite electrode with MWCNT, which was used as an anode in the OCV generation [3]. Each experiment was done in triplicate. The Microbial fuel cell electrical performance was examined and analyzed by oxidation and reduction peaks in cyclic voltammetry (CV) technique [4]. Sugar analysis in sample and byproduct analysis after MFC operation was done by HPLC using suitable mobile phase and concentration of each compound was determined [5].

**Results**

A fungal strain Isolated from soil showed both amylase and pectinase activity and molecular characterization of isolated strain was done which was found similar with Aspergillus niger. Potato peel waste had pH 6.51± 0.08, Total suspended solid (TSS) 21.4±1.27%, moisture content 78.6±1.26%, ash content 11.68±7.05%, volatile suspended solid (VSS) 88.31±7.056%, chemical oxygen demand (COD) 10.24±0.12 mg/g, reducing sugar 1.061±0.64 mg/g, ammoniacal-nitrogen 0.01±0.01 mg/g and phosphorus 0.015±0.017 mg/g. Similarly, potato peels contained iron 0.167mg/g, copper 0.007 mg/g, zinc 0.007 mg/g and manganese 0.005 mg/g but lead and nickel was not found. Open circuit voltage (OCV) generation was highest while using KMnO4 in catholyte (505±18 mV). Microbial fuel cell in fed batch was also performed by adding 10% of sample in every 24 h, and clearly improved result was obtained in this operation. Power density was determined using 100 ohm and 1000-ohm external resistors which was found 119±7 W/m3 and 42±9 W/m3 respectively. From Microbial fuel cell operation at optimized condition removal rate of COD, ammoniacal-nitogen, reducing sugar and total suspended solid were found to be 37.69%, 67.72%, 72.64% and 65.95% respectively. The Microbial fuel cell electrical performance was examined and analyzed by oxidation and reduction peaks in cyclic voltammetry (CV) technique. Sugar analysis in sample and byproduct analysis after MFC operation was done by HPLC from which concentration of glucose was found 0.87±0.06 mg/g of sample. Similarly, among all organic acids highest amount of citric acid was found (3.04±0.11 mg/g of sample).

**Conclusion**

Finally, this research conveys that the use of enzymes produced by fungal strain can be a novel alternative approach in bioelectricity production and is beneficial in management of solid waste also, as it does not require separate pretreatment.

**Acknowledgement**

We would like to thank Prof. Dr. Krishna Das Manandhar from Central Department of Biotechnology, Tribhuvan University, Kirtipur for his support during the research period.

**References**

1. Singh, B., Singh, J., Singh, J. P., Kaur, A., & Singh, N. (2020). Phenolic compounds in potato (Solanum tuberosum L.) peel and their health-promoting activities. International Journal of Food Science and Technology, 55(6), 2273–2281. https://doi.org/10.1111/ijfs.14361
2. Wang, C. T., Liao, F. Y., & Liu, K. S. (2013). Electrical analysis of compost solid phase microbial fuel cell. International Journal of Hydrogen Energy, 38(25), 11124–11130. 10.1016/j.ijhydene.2013.02.120
3. Abdulla, S., Mathew, T. L., & Pullithadathil, B. (2015). Highly sensitive, room temperature gas sensor based on polyaniline-multiwalled carbon nanotubes (PANI/MWCNTs) nanocomposite for trace-level ammonia detection. Sensors and Actuators, B: Chemical, 221, 1523–1534. 10.1016/j.snb.2015.08.002
4. Venkata Mohan, S., Mohanakrishna, G., & Sarma, P. N. (2010). Composite vegetable waste as renewable resource for bioelectricity generation through non-catalyzed open-air cathode microbial fuel cell. Bioresource Technology, 101(3), 970–976. https://doi.org/10.1016/j.biortech.2009.09.005
5. Bio-Rad. (2012). Aminex HPLC Columns ®. Bulletin 6333 Rev A US/EG, 7–10.

## A28. Isolation, antifungal activity, physiological features, and growth potential of native *Trichoderma* spp. on alternative substrates

### Rozina Giri^1, 2^, Sagun K.C. ^1, 2^, Sanju Tamang^1, 2^, Surakshya Singh^1, 2^, Nawanit Kumar Mahato1, Ashok Bhattarai^3^, Mitesh Shrestha^1, 2^

#### ^1^ Department of Applied Microbiology and Food Technology, Research Institute for Bioscience and Biotechnology, Kathmandu, Nepal; ^2^ Kantipur Valley College, Kumaripati, Lalitpur, Nepal; ^3^ Praramva Biotech Pvt. Ltd. Kathmandu, Nepal

##### **Correspondence:** Mitesh Shrestha (mitesh.shrestha@ribb.org.np)

*BMC Proceedings 2023*, **17(Suppl 3):**A28

**Background**

Biological Control Agents (BCAs) have recently gained huge traction owing to their potential for becoming a healthy and environment-friendly alternative to toxic chemical pesticides. Trichoderma is one of the most used BCAs owing to their global distribution and broad range of antagonism against various phytopathogens as well as plant-growth promoting activities. Owing to the geographical and climatic variability, it is useful to utilize the native local resources.

**Materials and methods**

*Trichoderma* spp. were isolated using serial dilution techniques from soil collected from various parts of Nepal. Dual culture was performed against phytopathogens. Effect of nitrogen on *Trichoderma* growth was checked. Similarly, the effect of Carbendazim was assessed for both Trichoderma as well as the phytopathogens. Finally, Trichoderma cultivation was done in various alternative substrates.

**Results**

Six native *Trichoderma* species present in soil from Chitwan, Dhapakhel, Godawari, Phulchowki, Pokhara and Simtal were isolated. Similarly, during dual culture against two phytopathogens *Fusarium oxysporum* and *Alternaria alternata*, strain from Pokhara showed the highest antagonistic activity of 62.71 % and 55.19 % respectively. There was a gradient effect of nitrogen on Trichoderma growth, with higher concentration inhibiting the growth. The fungicide Carbendazim inhibited all *Trichoderma* species as well as *Fusarium oxysporum* while had limited effect on *Alternaria alternata* at all used concentrations (0.1 %, 0.15 %, and 0.2%). Similarly, the mass multiplication of *Trichoderma* sp. on various alternative liquid (Potato Dextrose Broth, 3 % Molasses, and 20 % Whey) and solid (wheat, inner flesh of sugarcane bagasse, whole sugarcane bagasse, and rice husk) substrates showed excellent growth, with the highest growth observed in 20 % whey and rice husk.

**Conclusions**

Hence, we found that the isolated *Trichoderma* sp. could be grown utilizing low-cost, alternative agricultural byproducts.

## A29. Fluorescence properties of organic contamination in water

### Sishir Gautam^1^, Suvechhya Bastola^2^, Anusa Thapa^1^, Prashant Waiba^1^, Sanket Bohora^1^, Prajwal Rajbhandari^2^, Thomas Krauss^3^, Ashim Dhakal^1^

#### ^1^Phutung Research Institute, Kathmandu, Nepal; ^2^ Department of Applied Microbiology and Food Technology, Research Institute for Bioscience and Biotechnology, Kathmandu, Nepal ^3^ University of York, York, UK

##### **Correspondence:** Sishir Gautam (sishir.gautam@pinstitute.org)

*BMC Proceedings 2023*, **17(Suppl 3):**A29

**Background**

Increased anthropogenic inputs in the water body have dramatically changed the loads and compositions of dissolved organic matter (DOM) in urbanized streams, which has a negative impact on the water. As such, it is imperative to monitor water quality regularly. Existing methods for identifying the contaminant species are not only expensive and time consuming but also require skilled manpower, consumables and resources [1,2]. The interaction of light with DOM is a function of its chemical makeup; thus, fluorescence spectroscopy can provide information about the amount and type of DOM in a water sample. The purpose of this study is to explore fluorescence properties of water as a screening and continuous water quality monitoring technique, to develop a rapid low-cost water testing technology.

**Materials and methods**

Fluorescence signal from the water sample was measured using a PhotoMultiplier Tube (PMT) based fluorescence spectrometer. The setup consists of a UV LED (Thorlabs M275L4) light that excites the sample at 275 nm wavelength. The fluorescence from the sample is filtered by band-pass filters with the pass band at 350 +/- 30 nm and focused onto the PMT. The fluorescence from the other face of the cuvette is coupled to an optical fiber which guides the light to a CCD spectrometer that records an emission spectra.

**Results**

Humic-like fluorescence (HLF),420 nm peak, is a dominating fluorescence signal in the underground water while tryptophan-like fluorescence (TLF), 350 nm peak, is the dominating fluorescence in the urban Bagmati river water. Drinking water that is purified and sealed, has very little fluorescence without any TLF or HLF peak.

We also observed significant TLF signals from the organic compounds like amino-acids, starch, nucleic acid and alcohols. From this, we can infer that the fluorescence signal at 350 nm wavelength can also be used to track organic contamination in water.

**Conclusions**

TLF and HLF are indicators of organic matter contamination, which may be from sewage, farms, chemical spillage, and massive microbial activity in the water body. The relative simplicity of sampling and fluorescence analysis allows for quick monitoring of water quality.

**Acknowledgement**

This work was supported by EPSRC’s GCRF Grant# EP/T020008/1 & EP/W524165/1; TWAS & SIDA Grant # 18-013. Additionally, I would like to thank Phutung Research Institute’s Suman Basnet, Pravin Bhattarai, and Rijan Maharjan for helpful discussions.

**Reference**

1. Sorensen, J., Diaw, M., Pouye, A., Roffo, R., Diongue, D., Faye, S., Gaye, C., Fox, B., Goodall, T., Lapworth, D., MacDonald, A., Read, D., Ciric, L. and Taylor, R. In-situ fluorescence spectroscopy indicates total bacterial abundance and dissolved organic carbon. Science of The Total Environment. 2020;738: p.139419.
2. Ward J, Lapworth D, Read D, Pedley S, Banda S, Monjerezi M et al. Tryptophan-like fluorescence as a high-level screening tool for detecting microbial contamination in drinking water. Science of The Total Environment.2021;750:141284.

## A30. Genetic polymorphisms of genes involved in host immune response to dengue severity in Nepalese population

### Chetana Khanal, Sabita Prajapati, Machchhendra Thapa, Ramanuj Rauniyar, Krishna Das Manandhar

#### Central Department of Biotechnology, Tribhuvan University, Kirtipur, Nepal

##### **Correspondence:** Chetana Khanal (chetanakhanal12@gmail.com)

*BMC Proceedings 2023*, **17(Suppl 3):**A30

**Background**

In dengue, an endemic country like Nepal all four serotypes (DENV1-4) of dengue virus (DENV) is found to be prevalent causing dengue fever and its severe form dengue hemorrhagic fever (DHF) and dengue shock syndrome (DSS). The severity of the disease is determined by virus serotype, host immune, and genetic status which if known, can help in early disease management. The individual's genetic makeup differs from place to place around the world, which might be the reason for one population being more susceptible and the other being resistant to the virus. Study is focused on the severity marker prediction which might help in the early diagnosis of severe dengue cases

**Materials and methods**

A study on the severity-determining marker is being carried out in endemic areas in human samples, as there is no appropriate animal model mimicking the DHF/DSS as in humans. Genetic Marker was detected by Single nucleotide polymorphism detection by ARMS PCR.

**Results**

Statistical analysis of the clinical parameters of all the case samples was performed to classify the samples in dengue hemorrhagic (DHF) 53%, severe dengue (DS) 14%, and Dengue fever (DF)33%. Serotyping by Real-time and Nested PCR showed the co-circulation of DENV-2 in the year 2019. Single nucleotide polymorphism (SNP) of four different genes were identified using amplification refractory mutation system PCR using allele-specific primers for TNF-α (+308A/G), IFNG(+874A/T), IL-10(819C/T,1082 A/G) and FCgRIIa. CT genotype of TNF-α was observed in 60% of the case population, in the case of Interleukin-10(819 C/T,1082 A/G) CT and AG genotype was seen in most of the study population 71% and 62% respectively. For IFNG(+874A/T), AT(45%) genotype was followed by AA(40%) whereas only 15% with TT genotype was reported. In case of FCgRIIa, a heterozygote genotype AG variant was detected in all study populations.

**Conclusion**

As the population size was small, the role of SNPs in severity could not be exactly predicted but the study provides future insight into predicting the role of these biomarkers in susceptibility, progression, or protection of disease.

## A31. Mining envelope domain iii of dengue virus for recombinant tetravalent dna vaccine candidate from nepalese samples

### Machchhendra Thapa, Sabita Prajapati, Chetana Khanal, Ramanuj Rauniyar, Sishir Gautam, Tinmaya Rai, Krishna Das Manandhar

#### Central Department of Biotechnology, Tribhuvan University, Kirtipur, Nepal

##### **Correspondence:** Machchhendra Thapa (thapa.machchhendra@gmail.com)

*BMC Proceedings 2023*, **17(Suppl 3):**A31

**Background**

Dengue is a mosquito-borne infection caused by the Dengue virus (DENV) which leads to diseases in humans from mild dengue fever to severe Dengue. All four serotypes (1-4) of dengue viruses can infect humans. Once infected the risk of development of severe dengue during secondary infection due to Antibody Dependent Enhancement (ADE) increases which results in enhanced virus entry and greater virus replication. It is also one of the challenges in vaccine development and thus acceptance. So there is an urgent need for a dengue vaccine that induces long-lasting protection for all four serotypes of dengue while avoiding the immune enhancement of viral infection. The envelope region has been widely studied for vaccine development. Among Domain I, II, and III of the envelope region, Domain III remains the choice of interest due to its reduced risk of Antibody Dependent Enhancement (ADE) in dengue infection. Considering EDIII as a candidate instead of a whole envelope might address the solution to existing problems of dengue vaccine in use and it might eliminate the risk of ADE.

**Materials and methods**

In the present study, we aim to identify all four serotypes and amplify serotype-specific EDIII regions. Further, we aim to fuse the EDIII region of all four dengue virus serotypes to make a single recombinant tetravalent ED III dengue vaccine construct for its subsequent use as a novel vaccine candidate. Further, we aim to perform *insilico* analysis of the vaccine candidate.

**Results**

We amplified and sequenced the Domain III of serotype I and serotype II and retrieved the Domain III sequence of serotype III and serotype IV from the NCBI database. *Insilico* analysis of the tetravalent construct showed the protein consists of 400 amino acids with 11 epitopes present in the protein. And protein was classified as mixed protein.

**Conclusion**

Based on the analysis, the tetravalent construct can be a potential vaccine although EDIII represents only a fraction of the envelope protein, the removal of other epitopes that elicit non-neutralizing, cross-reactive antibodies should reduce the risk of developing ADE and disease progression to DHF or DSS.

## A32. Efficacy of biological treatments against root-knot nematode (*Meloidogyne* spp.) in okra (*Abelmoschus esculentus* L.) at Nawalparasi, Nepal

### Kritika Adhikari^1^, Gaurav Adhikari^1,2^, Susmita Sigdel^1^, Santosh Marahatta^1^

#### ^1^Agriculture and Forestry University, Rampur, Nepal; ^2^Department of Natural Products and Green Chemistry, Research Institute for Bioscience and Biotechnology, Kathmandu, Nepal

##### **Correspondence:** Gaurav Adhikari (gauravadhikari1997es@gmail.com)

*BMC Proceedings 2023*, **17(Suppl 3):**A32

**Background**

Root-knot nematode (*Meloidogyne* spp.) is an important soil-borne pathogen affecting several vegetable crops including Okra. A pot trial was carried out in the vegetable zone, Danda, Nawalparasi East from February to June 2021 to assess the management strategy against root-knot nematode in Okra var. Arka anamika under field conditions.

**Materials and methods**

The experiment was carried out in Randomized Complete Block Design with seven treatments replicated thrice. The treatment consisted of Neem cake, *Trichoderma viride,* Abamectin, *Pseudomonas fluorecsens*, *Trichoderma viride +* Spent Mushroom Substrate (SMS), A-Arya 009, and control.

**Results**

All the treatments showed significant results in percentage disease reduction (p < 0.001) compared to the control treatment. Abamectin (69.57%) was the most effective at reducing the root infection followed by neem cake (56.52%) *Pseudomonas fluorescens* (47.83%), *Trichoderma viride* + SMS (34.78%), and *Trichoderma viride* (34.78%) while the least reduction in disease was recorded in A-Arya 009 (17.39%). The highest yield advantage over control was recorded in Neem cake (45.45%) followed by abamectin (38.22%), *Trichoderma viride +* SMS (32.28%), *Pseudomonas fluorescens* (32.02), *Trichoderma viride* (31.67%), and the least yield improvement was recorded in A-Arya 009 (15.77%). A significant negative correlation (p < 0.001) was found between gall index and total yield implying heavy losses with the progression of root infection.

**Conclusion**

The study concluded that neem cake and Abamectin as an effective biological treatment for managing root-knot nematodes and improving the yield of Okra.

