# Publisher Correction: Harnessing a decade of research at the Research Institute for Bioscience and Biotechnology in Kathmandu, Nepal: Proceedings of the Fourth International Conference ICBB-2022

**DOI:** 10.1186/s12919-023-00282-x

**Published:** 2024-01-09

**Authors:** Suvechhya Bastola, Remco Kort, Prajwal Rajbhandari

**Affiliations:** 1 Department of Applied Microbiology and Food Technology, Research Institute for Bioscience and Biotechnology, Kathmandu, Nepal; 2 Amsterdam Institute for Life and Environment (A-LIFE), Vrije Universiteit Amsterdam, Amsterdam, The Netherlands

**Publisher Correction: BMC Proc 17, 3 (2023)**

**https://doi.org/10.1186/s12919-023-00252-3**

Several publisher's mistakes in affiliations of authors and body text.

Although authors informed the publisher, the following mistakes still occurred in the text of the article [1]. The list of the corrected parts of the text is mentioned as per below:

Page 1 — The RIBB was founded in 2011 with the mission to advance the fields of bioscience, engineering, innovation and education by collaborating with academic and other research organizations to conduct high quality research in a world-class academic and professional environment.

Page 2 — The fourth edition, the ICBB-2022, entitled ‘A decade in research: Celebrating RIBB’s 10th anniversary’, encompassed a wider variety of research themes.

Page 2 — Reference 6 should be: Majhi R, Maharjan R, Shrestha M, Mali A, Basnet A, Baral M, Duwal R, Manandhar R, Rajbhandari P. Effect of altitude and solvent on Psidium guajava Linn. leaves extracts: phytochemical analysis, antioxidant, cytotoxicity and antimicrobial activity against food spoilage microbes. BMC chemistry. 2023 Dec;17(1):1–6.

Page 2 — The proceedings ICBB-2022 included a total of 32 abstracts. These abstracts covered a wide range of research topics under each thematic session.

Page 2 — As organizers, we are immensely pleased that we were able to continue this conference to its fourth edition.

Page 3 — A1 Abstract “Workshop on public engagement program at school”.

Suvechhya Bastola1, 2, Alba Abad1, 3, Rojlina Manandhar4, Surakshya Singh4, Bibek Chandra Mahaseth4, Kamana Dawadi4, Lochan Pandeya4.

1 Engage Nepal with Science, Edinburgh, UK; 2 Learning Lab, Research Institute for Bioscience and Biotechnology, Kathmandu, Nepal; 3 Wellcome Centre for Cell Biology, University of Edinburgh, Edinburgh, UK; 4 Department of Natural Products and Green Chemistry, Research Institute for Bioscience and Biotechnology, Kathmandu, Nepal.

Page 4 — A3 RIBB’s 10th year journey: story presentation.

Page 14 — A28 Abstract “Isolation, antifungal activity, physiological features, and growth potential of native Trichoderma spp. on alternative substrates”.

Rozina Giri1, 2, Sagun K.C.1, 2, Sanju Tamang1, 2, Surakshya Singh1, 2, Nawanit Kumar Mahato1, Ashok Bhattarai, 3, Mitesh Shrestha1, 2.

1 Department of Applied Microbiology and Food Technology, Research Institute for Bioscience and Biotechnology, Kathmandu, Nepal; 2 Kantipur Valley College, Kumaripati, Lalitpur, Nepal; 3 Praramva Biotech Pvt. Ltd. Kathmandu, Nepal.

Page 14 — A29 Abstract “Fluorescence properties of organic contamination in water”.

Sishir Gautam1, Suvechhya Bastola2, Anusa Thapa1, Prashant Waiba1, Sanket Bohora1, Prajwal Rajbhandari2, Thomas Krauss3, Ashim Dhakal1.

1 Phutung Research Institute, Kathmandu, Nepal; 2 Department of Applied Microbiology and Food Technology, Research Institute for Bioscience and Biotechnology, Kathmandu, Nepal 3 University of York, York, UK.

Page 16 — A32 Abstract “Efficacy of biological treatments against root-knot nematode (Meloidogyne spp.) in okra (Abelmoschus esculentus L.) at Nawalparasi, Nepal”.

Kritika Adhikari1, Gaurav Adhikari1, 2, Susmita Sigdel1, Santosh Marahatta1.

1 Agriculture and Forestry University, Rampur, Nepal; 2 Department of Natural Products and Green Chemistry, Research Institute for Bioscience and Biotechnology, Kathmandu, Nepal.

The original was updated.
